# A multiplex inhalation platform to model *in situ* like aerosol delivery in a breathing lung-on-chip

**DOI:** 10.3389/fphar.2023.1114739

**Published:** 2023-03-06

**Authors:** Arunima Sengupta, Aurélien Dorn, Mohammad Jamshidi, Magali Schwob, Widad Hassan, Lea Lara De Maddalena, Andreas Hugi, Andreas O. Stucki, Patrick Dorn, Thomas M. Marti, Oliver Wisser, Janick D. Stucki, Tobias Krebs, Nina Hobi, Olivier T. Guenat

**Affiliations:** ^1^ Organs-on-Chip Technologies, ARTORG Center for Biomedical Engineering, University of Bern, Bern, Switzerland; ^2^ AlveoliX AG, Swiss Organs-on-Chip Innovation, Bern, Switzerland; ^3^ Department of General Thoracic Surgery, Inselspital, Bern University Hospital, Bern, Switzerland; ^4^ Department for BioMedical Research, University of Bern, Bern, Switzerland; ^5^ VITROCELL Systems GmbH, Waldkirch, Germany; ^6^ Department of Pulmonary Medicine, Inselspital, Bern University Hospital, Bern, Switzerland

**Keywords:** lung-on-chip, inhalation therapeutics, nanoparticles, aerosolized drug delivery, cyclic stretch, air-liquid interface, COPD, toxicity assessments

## Abstract

Prolonged exposure to environmental respirable toxicants can lead to the development and worsening of severe respiratory diseases such as asthma, chronic obstructive pulmonary disease (COPD) and fibrosis. The limited number of FDA-approved inhaled drugs for these serious lung conditions has led to a shift from *in vivo* towards the use of alternative *in vitro* human-relevant models to better predict the toxicity of inhaled particles in preclinical research. While there are several inhalation exposure models for the upper airways, the fragile and dynamic nature of the alveolar microenvironment has limited the development of reproducible exposure models for the distal lung. Here, we present a mechanistic approach using a new generation of exposure systems, the Cloud α AX12. This novel *in vitro* inhalation tool consists of a cloud-based exposure chamber (VITROCELL) that integrates the breathing ^AX^Lung-on-chip system (AlveoliX). The ultrathin and porous membrane of the AX12 plate was used to create a complex multicellular model that enables key physiological culture conditions: the air-liquid interface (ALI) and the three-dimensional cyclic stretch (CS). Human-relevant cellular models were established for a) the distal alveolar-capillary interface using primary cell-derived immortalized alveolar epithelial cells (^AX^iAECs), macrophages (THP-1) and endothelial (HLMVEC) cells, and b) the upper-airways using Calu3 cells. Primary human alveolar epithelial cells (^AX^hAEpCs) were used to validate the toxicity results obtained from the immortalized cell lines. To mimic *in vivo* relevant aerosol exposures with the Cloud α AX12, three different models were established using: a) titanium dioxide (TiO2) and zinc oxide nanoparticles b) polyhexamethylene guanidine a toxic chemical and c) an anti-inflammatory inhaled corticosteroid, fluticasone propionate (FL). Our results suggest an important synergistic effect on the air-blood barrier sensitivity, cytotoxicity and inflammation, when air-liquid interface and cyclic stretch culture conditions are combined. To the best of our knowledge, this is the first time that an *in vitro* inhalation exposure system for the distal lung has been described with a breathing lung-on-chip technology. The Cloud α AX12 model thus represents a state-of-the-art pre-clinical tool to study inhalation toxicity risks, drug safety and efficacy.

## Introduction

Inhalation is a major route of airborne toxicant exposure, in which the lung epithelial barrier serves as the main portal of entry into the systemic circuit for the human body. This may lead to progressive and perpetual inflammation of the epithelium leading to development of chronic respiratory conditions such as asthma, COPD, ARDS and interstitial lung diseases (ILD) (Baumgartner et al., 2000; Sokol et al., 2013; Assad et al., 2018; Zhao et al., 2019; Peng et al., 2020; Shima et al., 2022). Inhaled toxicants are ubiquitous across the globe and come from a variety of sources ranging from organic pollution to industrial chemicals and consumer-related products (Adeola, 2021; Naidu et al., 2021). Therefore, it is crucial to understand the harmful effects of these inhaled substances, especially in the lungs, being the primary point of contact. Inhaled toxicants are known to trigger microinjuries which lead to epithelial barrier damage (Carlier et al., 2021), activation of alveolar macrophages (AMs) (Hamilton et al., 2008; Kuroda et al., 2016) and differentiation and translocation of the circulating monocytes into the alveolar space (van Eeden et al., 2001; Bliss et al., 2018).

To assess the impact of inhaled substances, human exposure data are still considered as the “gold standard” to identify and understand potential risk factors. However, controlled human exposure and epidemiologic studies for respirable substances are generally very costly, of long duration and when suspected to be toxic, non-ethical (London et al., 2010). Therefore, toxicity tests have mainly been carried out in animals, typically rats to fill this knowledge gap. . Inhalation testing using animals is still a regulatory requirement in most jurisdictions including Canada, the United States of America (United States of America), and the European Union (E.U.) for substances such as chemicals, plant production products or pharmaceuticals (Stucki et al., 2022). However, due to scientific and ethical concerns, and to adhere to the 3R principles (Ehrhardt & Kim, 2008) to reduce, refine and replace the use of animals in science, regulations worldwide are being amended and new approaches are being developed.

For example, the Senate of Canada passed the “Bill S-5” in June 2022 (Bill S-5.44-1, 2022), an act to amend the Canadian Environmental Protection Act to strengthen the importance of alternatives to animal testing. The FDA Modernization Act 2.0 was signed into U.S. law by President Biden in December 2022, amending and deleting wording from 1938 in the Food, Drugs, and Cosmetics Act that required all drugs to be tested on animals before conducting clinical trials (Animal Wellness Action, 2022). In addition, the U.S. Food and Drug Administration (FDA) has also set out new proposals for its New Alternative Methods Programs in June 2022 (Nuwer, 2022). In November 2022, the European Commission and European Chemicals Agency announced its commitment to modernize alternative non-animal *in vitro* testing strategies and to develop a roadmap to phase-out animal testing (EPAA, 2022). Furthermore, international efforts are going on to develop robust and efficient processes to establish scientific confidence in new approaches and to facilitate the timely uptake into regulatory application of alternatives to animal testing (van der Zalm et al., 2022).

Several alternative *in vitro* approaches for inhalation toxicity testing have been developed in the past years (Clippinger et al., 2018; Singh et al., 2021). Until a few years ago, most inhalation testing with these approaches were typically performed by exposing the cells or tissues to the substance by pipetting (Ramanarayanan et al., 2022; OECD case no. 367). However, previous studies have demonstrated that nebulization of chemicals and drugs to mimic realistic aerosol exposure has resulted in faster (about 12 folds higher) uptake of inhaled compounds (Lenz et al., 2014). Also, observations made by comparing transepithelial barrier (TER) measurements, oxidative stress and cytotoxic effects of nebulized compounds in air-liquid interface (ALI) correlated better with *in vivo* readouts (Fizeșan et al., 2018; Stewart et al., 2012; H; Wang H. et al., 2018) compared to submerged cells where the compound is instilled. Moreover, submerged cell cultures lack proper physiological representation because aerosols generally sediment on the air-filled bronchial and alveolar epithelium (Fröhlich et al., 2013). The polarity of the epithelial cells and the presence of mucus or surfactant (SP) on the cell layer also plays a vital role in simulating the *in situ* environment of the epithelium which is only achieved through ALI culture conditions (Hobi et al., 2012; Schleh et al., 2013; Lock et al., 2018). Furthermore, multicellular co-culture models contribute to make particle translocation models more predictable and realistic (Rothen-Rutishauser et al., 2005). In particular, alveolar epithelial cells covering the distal airspace act as a gateway keeper for the systemic circulation of inhaled particles. Hence, a complex mixture of AT1 and AT2 cells is required to accurately model the alveoli *in vitro* (Sanches-Zuttion et al., 2022). In addition, immune cells like macrophages play a vital role for triggering a proper inflammatory cascade in response to infection (Artzy-Schnirman et al., 2021) as well as nebulized toxicants (Ji et al., 2018) in the alveoli.

Although ALI cell cultures capture the complicated nature of the lung epithelium with a high degree of precision, yet the slow development of inhalation therapy reflects the lack of efficient aerosol delivery systems compatible with medium to high throughput biomimetic technologies. Rats and mice have been popularly used as surrogate animals so far for lung inhalation *in vivo* (Kolanjiyil et al., 2019; S. Y; Lee et al., 2008; Lewis et al., 2013) and *ex vivo* (Hess et al., 2016; Cidem et al., 2020) studies. The lungs with its exclusive functionality provide a unique opportunity to target it directly *via* inhalation therapy and thus reduce systemic side-effects. However, the small number of approved inhaled drugs and in general the very high attrition rate of respiratory drugs (Barnes et al., 2015) shows the difficulty of developing such drugs as well as the lack of standardized predictive pre-clinical models. One of the major limitations for developing inhalation exposure system is the substantially higher amount (30 folds) of expensive testing drug requirement (where 10 mg/kg in oral = 1.7 g v/s approximately 50 g for inhalation) in efficacy studies (Resources, Charles River 2022). Moreover, until now, the pharma companies were not so much invested in inhaled therapy, therefore the lack of interest and demand has substantially affected the growth of preclinical tools for studying inhaled medicines. As of today, “inhaled therapeutics” have gained considerable momentum, hence current research trends focus on the development of advanced *in vitro* (Abaci & Shuler, 203315) and *in silico* platforms (Antonio et al., 2016; Walenga et al., 2019) to address the limitations with animal models. These advanced *in vitro* models are able to provide reliable tools that combine human-relevant inhalation exposure systems including particle diffusion and sedimentation kinetics with *in-vivo*-like features of the lung epithelium barrier, macrophage-assisted clearance, multicellular communication and differentiation (He et al., 2022).

Earlier, traditional cell exposure devices used ultrasonic or jet nebulizers (Schreier et al., 1998; Bur et al., 2009; Cooney & Hickey, 2011) which required longer experimental duration, complicated handling and most importantly had poor aerosol-to-cell ratio. However, modern cloud-based exposure solutions employ vibrating mesh nebulizers that have overcome these limitations and are efficiently delivering aerosols to cells in ALI cultures (Lenz et al., 2014). Several research groups have successfully utilized commercially available systems like Vitrocell-CLOUD 6 and 12 (VITROCELL Systems GmbH) to predict the fate of aerosolized nanoparticles on transwell ALI cell cultures (Leibrock et al., 2019; Ding et al., 2020; Leroux et al., 2022). These systems have also been used to develop screening platforms for inhaled protein formulations (Röhm et al., 2017) and profile toxic aerosolized biocidal agents like Carbendazim (Tollstadius et al., 2019). Although these advanced transwell ALI exposure models can be well used for upper airways, they are not suited to model the dynamic microenvironment of the distal alveolar space where cyclic stretch plays an imminent role in alveolar cell differentiation and lung disease progression (Desai et al., 2008; Wu et al., 2018; Sehlmeyer et al., 2020). Exposing lung cells to ALI culture conditions along with physiological levels of cyclic stretch has been recently established to affect particle uptake (Doryab et al., 2021), maintain alveolar characteristics (Sengupta et al., 2022) and contribute to lung inflammation (Wu et al., 2013). However, due to the lack of proper exposure systems, the relevance and importance for including cyclic stretch (CS) with ALI culture conditions has not been studied in depth for inhalation studies.

With the rapid advances in microfluidics, lung-on-chip platforms offer the possibility of integrating biologically relevant cell microenvironment including a soft extracellular matrix (ECM) substrate, bio-relevant breathing motions and flow conditions (Huh et al., 2010; Stucki et al., 2015; Stucki et al., 2018; Zamprogno et al., 2021). Recently an airway-on-chip model with an integrated bronchial tree was utilized to successfully predict *in situ* deposition outcomes for inhaled airborne particles (about 2 µm in size) in the upper respiratory tract. The authors reported significant increase in cytotoxicity along with reduced expression of the tight junction protein, Zonula Occludens 1 (ZO-1) after exposure to the streamed aerosol particles (Fishler et al., 2015; Elias-Kirma et al., 2020). Moreover, recent advances in airway on-chip technologies have led to the development of models that mimic chronic lung injuries. Specifically, two studies have used on-chip models to replicate the effects of cigarette smoke-induced lung injury (Benam et al., 2016) and electronic cigarette or vaping product-associated lung injury (Kaiser et al., 2021). These studies demonstrate the relevance and potential of microfluidic technology in reproducing *in vivo* conditions and studying the mechanisms of chronic lung injuries in a controlled and precise manner. There are a few advanced lung-on-chip models for the lower airways that are currently available which can recapitulate crucial features of the native alveolar *in vivo* milieu like topography (Huang et al., 2021) and breathing motion (Huh et al., 2010; Stucki et al., 2018; Sengupta et al., 2022). However, adequate inhalation-based lung-on-chip models for the distal lung are largely missing (Artzy-Schnirman et al., 2021).

Occupational exposure to nanosized titanium dioxide (TiO2) and zinc oxide (ZnO) NPs has been reported to cause severe lung toxicity, inflammation, and lead to emphysema development *in vivo* in animal studies and in human volunteers (Chen et al., 2006; Gustafsson et al., 2011; Monsé et al., 2019; Abdulnasser et al., 2020). Lung cells exposed to these NPs have also resulted in toxicity and inflammatory cytokines production (Remzova et al., 2019; Freire et al., 2021). For toxic chemical inhalation applications, a compound used in humidifier disinfectant, polyhexamethylene guanidine (PHMG) was used. PHMG inhalation was recently established in several studies to induce serious lung toxicity and fibrosis development (Kim et al., 2015; Park J. et al., 2019). To negate the inflammation caused by PHMG induction, a corticosteroid, fluticasone propionate (FL) was used. FL is included within the treatment regimen for severe COPD and asthma cases (Molino et al., 2018; NHS-choices. uk). Therefore, to establish a realistic anti-inflammatory drug treatment course, FL was included in our studies after PHMG-induction.

**FIGURE 1:**
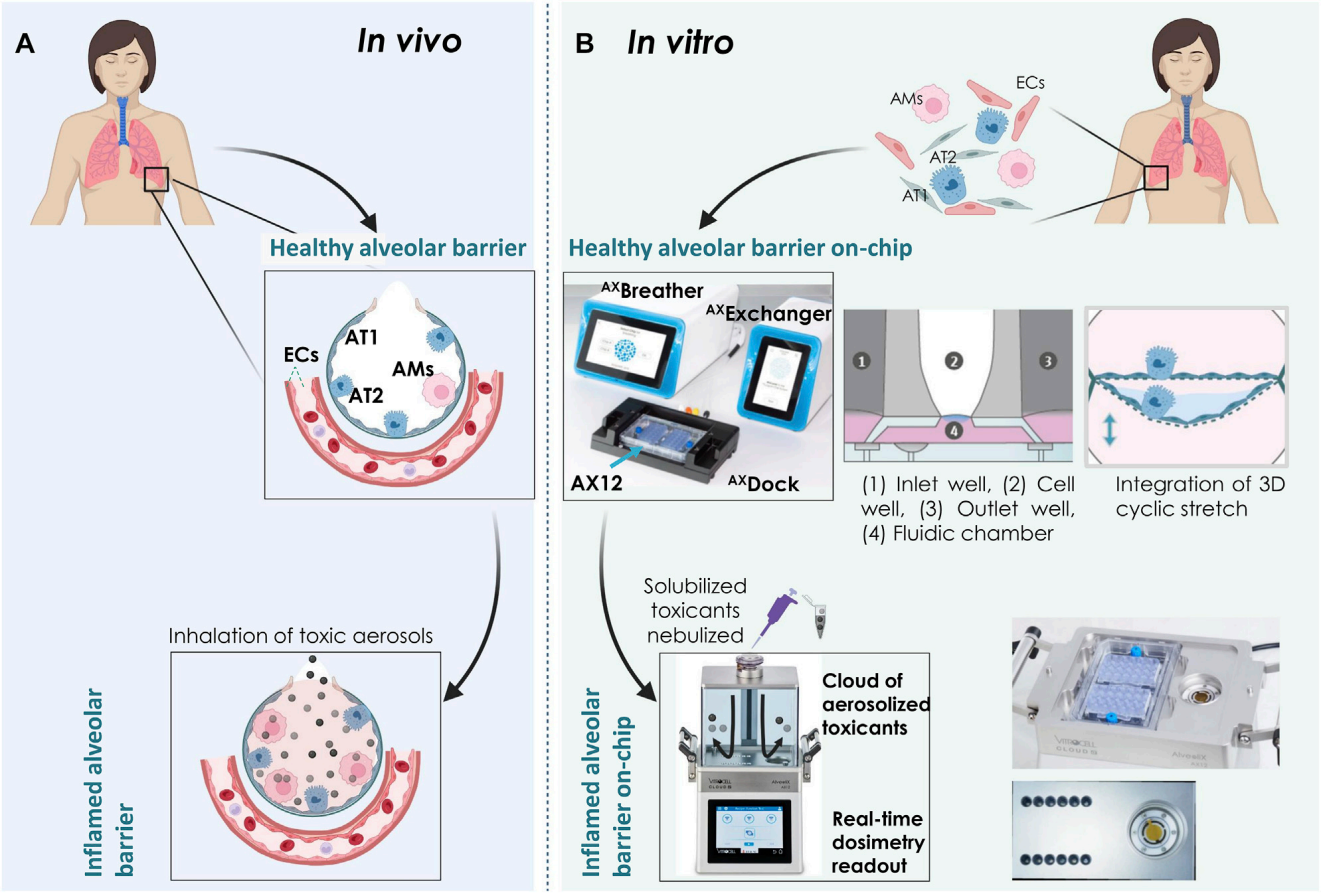
Overview of the Cloud α AX12 platform **(A)** An illustration showing the alveolar barrier *in situ*. The alveolar epithelial cells are situated on a thin basement membrane and are in close contact with the endothelial cells (ECs) on the basal side. The alveolar epithelium is comprised of squamous and thin alveolar type 1 (AT1) and cuboidal alveolar type 2 (AT2) cells along with resident alveolar macrophages (AMs). Inhaled toxic particles are represented as black and grey spheres. Inhalation of such airborne toxic particles result in lung inflammation, toxicity and progression to COPD. **(B)** Schematic representation of the ^AX^Lung-on-chip (including ^AX^Breather, ^AX^Exchanger, ^AX^Dock platform and the lung-on-chip consumable AX12) and Cloud α AX12 platform to model healthy and inflamed alveolar barrier *in vitro*. Primary and immortalized cells isolated from human sources were used in the model. Inflammation on-chip was mimicked using nebulized compounds with the Cloud α AX12. *Created with BioRender.com.*

We report here a next-generation inhalation *in vitro* platform (Cloud α AX12) that seamlessly integrates the cutting-edge breathing ^AX^Lung-on-chip technology with a cloud-based aerosol exposure chamber (Figure 1) to assess relevant physiological outcomes after toxicant or drug inhalation. Homogeneous and consistent cloud generation and aerosol dose distribution on-chip were initially tested using fluorescent tracer molecules. Three proof-of-concept studies (Table 1) were carried out with this system to determine a) NPs-induced inflammation (TiO2 and ZnO) in ALI and ALI + CS co-cultures on-chip, b) inhaled chemical inflammation and cytotoxicity triggered by PHMG, and c) inhaled corticosteroid treatment (FL) to reduce PHMG-induced inflammation. Given the unmet need for bio-relevant aerosol exposure setup for the alveolar space, the Cloud α AX12 represents a valuable tool for inhalation toxicology and drug safety and efficacy testing.

**TABLE 1:** Choice of environment-related substances used in the study.

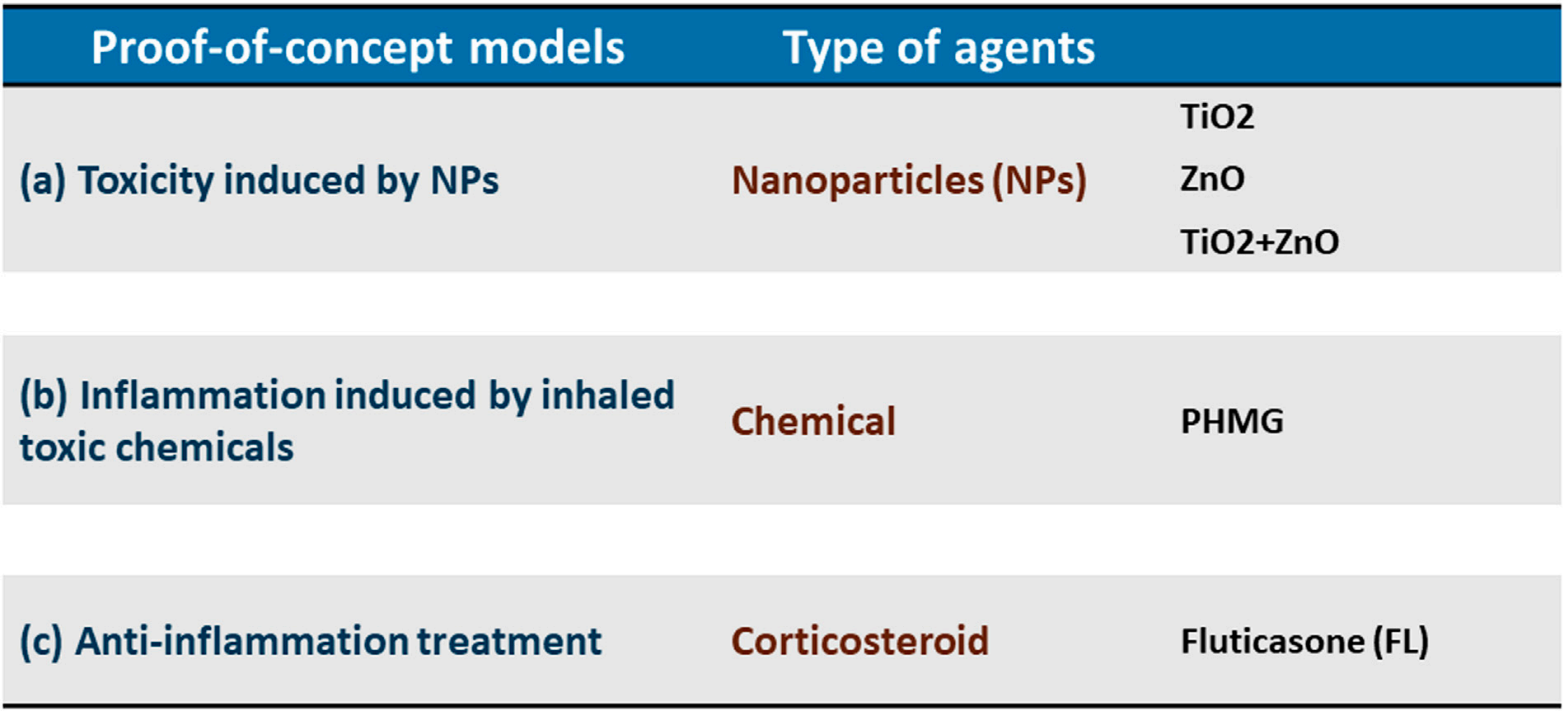

## Methods

### ^AX^Lung-on-chip

As described previously in Stucki et al., 2018 and Sengupta et al., 2022, the ^AX^Lung-on-chip system (AlveoliX AG, Switzerland) comprises of the Lung-on-Chip (AX12) consumable along with two electro-pneumatic devices (^AX^Exchanger and ^AX^Breather), and the interface platform (^AX^Dock) (Figure 1B). Cell seeding and medium exchanges were performed as recommended by the manufacturer (AlveoliX AG). To initiate the breathing mechanism, AX12 plate is positioned within the AXDock within the incubator. Then, three-dimensional (3D) CS (8% linear strain, 0.2 Hz) is initiated through a touchscreen control on the AXBreather, which deflects the microdiaphragm by generating negative cyclic pressure. CS and non-CS conditions are possible to run simultaneously on one AX12 plate, as the two chips are controlled independently.

### Operation of the cloud α AX12

The Cloud α AX12 was used to perform the nebulization of chemicals and NPs on cells cultured inside the AX12, consumable of the ^AX^Lung-on-chip.

**FIGURE 2:**
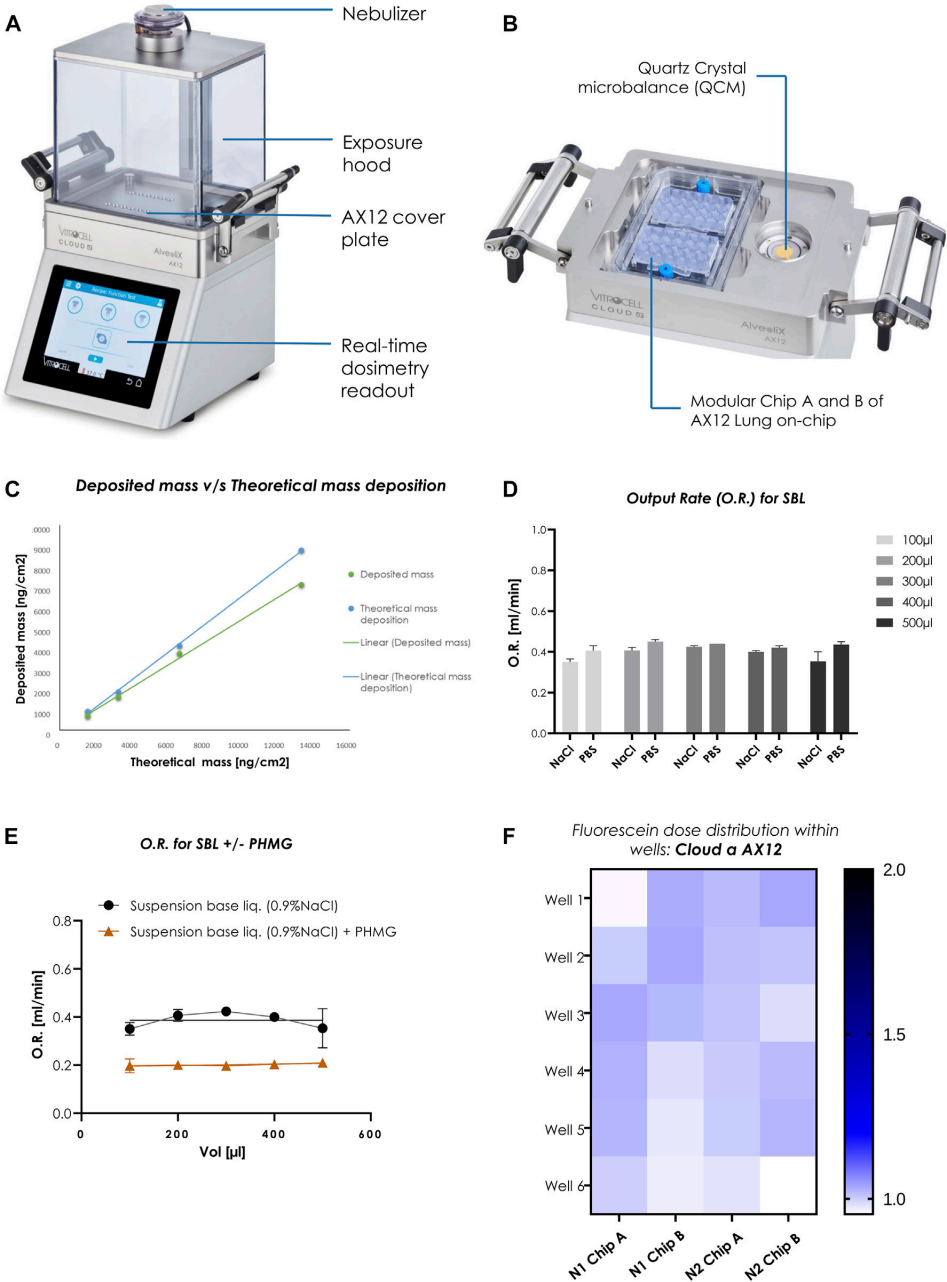
Homogenous dose distribution using the Cloud α AX12 **(A)** Design of the Cloud α AX12 comprised of a stainless-steel base module maintained at 37°C containing the AX12 plate placed on top covered by a steel plate. The holes on the steel base-plate are aligned to the wells within the AX12 plate. The QCM 6 placed inside the polycarbonate removable exposure hood quantify real-time cell delivered dose and are recorded and analyzed with the Vitrocell software. The nebulizer on top relies on a piezoelectric vibrating mesh to form a consistent aerosol cloud from the solubilized substance nebulized. **(B)** Detailed closer inspection of the orientation and placement for the AX12 plate and the QCM 6 sensor. **(C)** Comparison between deposited mass as measured from the QCM 6 readings (in green) and theoretically calculated (in blue). **(D)** O.R. (mL/min) was measured for different volumes (100–500 µL) for two SBLs (0.9% NaCl in distilled water and 1% PBS in distilled water) (n = 3). Data shown as mean ± SEM. **(E)** PHMG compound was dissolved in NaCl suspension base liquid as a reference. O.R. was measured for SBL without and with compound dilution using the Cloud α AX12 (n = 3). **(F)** Dose distribution measured from fluorescence signals [A.U.] of nebulized Fluorescein deposited on-chip. Signals from each well depicted in color scheme (white to blue; low fluorescence signal to high) from two individual rounds in AX12 plates (N = 2; n = 12).

The Cloud α AX12 uses a liquid aerosol nebulizer (Aerogen, Ireland) to produce cloud aerosol from solutions or suspensions. The nebulizer relies on a vibrating mesh technology (VMT) to form the aerosol (Figure 2A). Hereby, the nebulizer generates a high-frequency mechanical vibration from a piezoelectric element. This vibration is then transmitted to the mesh, a thin perforated membrane in contact with the solution. The vibratory movement of the mesh then creates a micro-pumping action that expulses droplets through the apertures (normally 4–6 μm in diameter, but other size ranges are available) and generates the aerosol. In the exposure chamber of the Cloud α AX12, the generated aerosol settles according to the principles of cloud settling and single droplet sedimentation. The cloud gets equally diverted and forms a symmetric pattern of vortices, to establish a spatially uniform sedimentation near the bottom of the chamber (Lenz et al., 2009). A homogeneous deposition is a crucial parameter to receive reproducible and consistent exposures of cells cultured in the AX12 plate under ALI conditions (Figure 2B).

A quartz crystal microbalance (QCM 6, VITROCELL, Germany) is integrated into the Cloud α AX12 allowing precise measurement of the deposited mass at the bottom of the exposure chamber (Ding et al., 2020). The QCM 6 consists of a piezoelectric quartz crystal oscillating at its resonance frequency by an electric oscillator (Figure 2B). The typical deposition profile was recorded by the QCM 6 during each nebulization step (Supplementary Figure S1A). The deposition profile can be divided into three phases (as described before in Ding et al., 2020): Phase I) Nebulization Phase: the nebulizer is activated and a dense cloud rapidly forms in the exposure chamber. The first droplets are deposited on the QCM 6 surface, leading to an increase of the QCM 6 signal. Phase II) Deposition Phase: 300 µL of the liquid suspension is completely nebulized and the cloud gradually settles to the bottom of the exposure chamber resulting in a steep increase in the QCM 6 signal. Next, the deposition signal reaches a maximum and the evaporation rate starts to increase, significantly reducing the QCM 6 signal and causing a plateau phase in which the saturated air inside prevents evaporation and stops aerosol deposition. Phase III) Evaporation Phase: the exposure chamber is opened and the QCM 6 is allowed to dry completely. This causes an abrupt rise in the QCM 6 signal, which stabilizes to the “true” mass deposition on the QCM 6 surface after a few seconds. It is important to note that only at this stage the QCM 6 signal represents the true deposited mass. Since the deposition values are measured at the end of evaporation phase for the toxicants, it is crucial that the QCM 6 signal remains stable over time since a shift in the baseline would affect reproducibility. Therefore, the baseline signal of the QCM6 must be verified. After 5 min (mins) (300 s) of recording, the signal of the QCM 6 was consistent across three different experiments (average SD: 21.3 ng/cm2) (Supplementary Figure S1B).

Before each experiment the exposure chamber was cleaned with 70% EtOH and heated to 37°C. To remove residues from previous usage, the nebulizer was dismounted and thoroughly cleaned according to the user manual. To pre-condition the exposure chamber, 300 μL of the suspension base liquid (SBL) (either 1% PBS or 0.9%NaCl) was nebulized. After cleaning, the AX12 was placed inside the heated compartment at the bottom of the exposure chamber as shown in Figure 2B. The AX12 is protected by a cover to ensure that the aerosol is deposited only within the cell wells and not on the remaining surfaces of the chip. Before starting the nebulization with the test substance, the exposure chamber was closed, and the microbalance zeroed. The nebulization was initiated by a touchscreen control on the Cloud α AX12. After the initiation of the nebulization, the cloud settled for 5–6 min. Hence the QCM 6 cannot measure liquid mass, it is important that only solid deposits remain (Ding et al., 2020). Therefore, the exposure chamber was then opened to evaporate the remaining liquid from the QCM 6 surface. Since deposition measurements usually take 5–10 min it is crucial to check that the QCM 6 signal does not drift during this period (Supplementary Figure S1B).

### Dose distribution measurements in cloud α AX12

To check consistent depositions in the cell wells, initial experiments were performed with a reference substance (fluorescein) measuring deposition with the QCM 6. Fluorescein is a water-soluble fluorescent tracer (fluorescein sodium salt, SIGMA, F6377-100G). To determine the accuracy of the QCM 6, different concentrations of fluorescein (25.00, 12.50, 6.25, 3.13, 1.56, and 0.78 mg/mL) were dissolved in saline solution (0.9% NaCl) and nebulized. The aerosol output rate (O.R.), in mL/min, was then calculated by measuring the time needed for the whole solution to pass through the mesh nebulizer.

Ideally, the entire nebulized suspension reaches the bottom of the exposure chamber where the cells are placed. This ratio is referred to as deposition factor (Eq. 1):

#### Deposition factor (f_dep_)

*V*_*neb*_ *represents the nebulized liquid volume and V*_*dep*_ *refers to the liquid aerosol volume deposited at the bottom of the exposure chamber (base steel plate).*

$$f_{dep}=\frac{V_{dep}}{V_{neb}}$$ (1)

300 μL from each fluorescein concentrations were nebulized consecutively. Additionally, 300 μL of pure PBS was nebulized as a control. The deposition factor was based on the fluorescein dose obtained from the QCM 6 (exposed area of the quartz crystal: 3.8 cm^2^). According to the nebulization protocol, the QCM 6 surface was rinsed with 300 μL 1X PBS in order to collect residual fluorescein. The fluorescence intensity result was then measured by plate reader type, absorption, wavelength (excitation wavelength: 483 nm; emission wavelength: 525 nm) and extrapolated to the entire surface area of the exposure chamber (200.75 cm^2^). Between each fluorescein nebulization, the nebulizer mesh was washed with 300 μL 1X PBS solution to avoid any bias in subsequent depositions. Finally, by comparing the fluorescence intensities between different exposed wells, the spatial uniformity of the aerosol deposition was assessed. For the evaluation of the theoretical mass deposition, the V_neb_ plays a role as described in Eq. 2:

#### Theoretical mass deposited (m)

*V*_*neb*_ *represents the nebulized liquid volume, m signifies the expected mass deposited in µg and c is the initial concentration (in µg/mL) of the test substance added to the nebulizer.*

$$m=c*V_{neb}$$ (2)

Additional experiments were performed to check the stability of the QCM 6 signal over time. Since exposure experiments typically last 5–10 min, it is important to check whether the QCM 6 idle values do not drift. To this end, the QCM 6 was gently and thoroughly wiped with an ethanol wetted tissue and the exposure chamber was warmed to 37°C. Once the QCM 6 was dry, the chamber was closed, and the microbalance was reset to 0. No substances were nebulized in this experiment; only the values given by the microbalance were recorded for 9 min (Supplementary Figure S1A). The sequence of a nebulization experiment using Fluorescein followed. The exposure chamber was opened after 5 min, and 2 more mins were recorded to mimic the drying phase of the QCM 6. Between each run the QCM 6 and the chamber were cleaned with a tissue soaked in 70% EtOH like for a normal nebulization experiment.

### Cell culture

The alveolar epithelial cell line (^AX^iAECs) used in this study was procured from AlveoliX (Switzerland). It was derived from primary human alveolar epithelial cells (AECs) that were isolated from resected lung tissue, and subsequently immortalized through the application of InscreeneX’s CI-Screen technology (described in Lipps et al., 2018). This cell line represents a valuable tool for *in vitro* toxicity studies, as it is derived from primary human cells and retains some of their characteristics. These cells were cultured according to manufacturer instruction as previously described and characterized (Sengupta et al., 2022).

Human primary alveolar type II cells (^AX^hAEpCs) were procured from AlveoliX (Switzerland). These cells were freshly isolated from lung tissue resections obtained with patient’s informed consent and ethical approval.

On AX12 plates, the ^AX^iAECs were maintained in AX Alveolar Epithelial Barrier medium supplemented with 1% penicillin-streptomycin (Thermo Fischer Scientific, Switzerland) and the ^AX^hAEpCs in AX Alveolar Epithelial Medium (AlveoliX, Switzerland). After closing the chips, wells were filled with the respective mediums using the initial feed option. For mono-culture studies ^AX^iAECs and ^AX^hAEpCs were seeded on the apical side of the membrane at a density of 4 × 10^5^ cells per cm^2^ in medium. ^AX^iAECs or ^AX^hAEpCs apical cell seeding is always considered as day 0 in all the studies.

Calu3 cells purchased from ATCC^®^ (Germany) was used as a human bronchial epithelial cell line. These cells were maintained in DMEM F-12 (Gibco; 21127-022) medium supplemented with 1% L-Glutamate, 10% fetal calf serum and 1% penicillin-streptomycin. For mono-culture studies Calu3 cells were seeded on the apical side of the membrane at a density of 1.9 × 10^5^ cells per cm^2^ in medium.

The THP-1 monocytes (ATCC; TIB-202) are immortalized monocyte-like cell line, derived from the peripheral blood of an acute monocytic leukemia case. These cells were cultured in RPMI (21875-034, Gibco) medium supplemented with 10% fetal calf serum and 1% penicillin-streptomycin in flasks. THP-1 monocytes were differentiated into macrophage-like cells using 8 nM and 200 nM phorbol 12-myristate 13-acetate (PMA; Sigma) and incubated for 48 h h) following previously established protocol (Kletting et al., 2018). Following incubation, the differentiated THP-1 (d-THP1s) macrophages were allowed to rest in PMA-free medium for further 24 h prior to seeding on-chip. For co-culture (^AX^iAECs/d-THP1s; 5:1 cell density ratio) experiments on-chip, the d-THP1s were added apically in equal parts of AX Alveolar Epithelial Barrier and RPMI medium.

Human Lung Microvascular Endothelial Cells (HLMVECs) purchased from PromoCell were expanded in flasks using AX Endothelial Medium. For the triple-cell culture model on-chip (^AX^iAECs/d-THP1s/HLMVECs; 5:1:2 cell density ratio), equal parts of AX E2 Alveolar Barrier Medium with RPMI were used. The HLMVECs were seeded on the basolateral side of each membrane, followed by a 2 h incubation and then by apical ^AX^iAECs seeding on day0. Cells were then maintained at 37°C, 5% CO_2_, and the medium was replaced every 2 days.

All cell lines were routinely monitored for *mycoplasma* contamination using a *mycoplasma* detection kit (MycoStrip, InvivoGen).

### TER measurement

To determine barrier formation, transbarrier electrical resistance (TER) measurements were taken every 2 days, starting 48 h after cell seeding as discussed previously (Sengupta et al., 2022). In brief, a commercially available 96-well plate electrode (STX100MC96; World Precision Instruments) and an Epithelial Volt/Ohm Meter (EVOM3; World Precision Instruments) was used. TER was measured in mono, co-culture and triple-cell culture conditions for up to 25 days. Before taking a reading, the electrodes were carefully sterilized using 70% v/v ethanol for 5 min and then rinsing them in distilled water for another 5 min at room temperature (RT). The “TER measurement” option was initiated on the AX Exchanger while taking readings. For cells kept in ALI, pre-warmed 1X sterile PBS was added 15 min prior to the measurements and then later re-equilibrated after TER readings. The background TER (ohm) data was obtained from a porous membrane having no cells. For analysis, the background subtracted TER (ohm) values were multiplied by the surface area of each cell culture well (0.071 cm^2^ on-chip) to obtain the final TER reading in ohm-cm^2^.

### Cloud α AX12 exposures

The Cloud α AX12 Exposure System (developed by Vitrocell GmbH. and AlveoliX AG.) was used for exposure of cell cultures to control nebulized aerosols at ALI. ZnO NPs (uncoated NM-110 from the European Commission’s Joint Research Centre - JRC; Ding et al., 2020) and TiO2 NPs (NM-105, CAS 13463-67-7) were kindly provided by Vitrocell. All NPs were diluted in 0.9% NaCl solution with a final volume of 300 µL. Prior to nebulization all NP-suspensions were sonicated in an ultrasonic bath (VWR #142-0084) for 15 min at 37°C to prevent agglomeration. Next, 100 μg/mL of the initial concentration of ZnO NPs were added to the nebulizer. Unless specified otherwise, high concentration studies of TiO2 NP, employed 100 μg/mL as initial concentration. The actual dose deposited on cells was calculated from the QCM 6 readings as 0.21 μg/cm^2^ for both ZnO and TiO2 NPs. For experiments with combination of NPs, 50 μg/mL of ZnO NPs and 50 μg/mL of TiO2 NPs initial concentration (final deposited concentration: 0.10 μg/cm^2^ TiO2 and 0.10 μg/cm^2^ ZnO) were added for nebulization. Exposure to NPs was performed always at day 21.

PHMG (Boc Sciences #CAS 89697-78-9) was diluted in 0.9% NaCl solution to a final volume of 300 µL to receive an initial concentration of 1.84 mg/mL. A final deposition of 2.7 µg/cm2 was measured on the cells. Fluticasone propionate (Fl; GlaxoSmithKline, #F9428) corticosteroid was diluted in 0.5% DMSO in sterile 1X PBS. Treatment experiments with Fl in 96-well plate were performed with initial concentrations ranging from 100 to 1000 nM (final deposition concentrations from 0.0747 to 0.747 µg/cm2). On-chip, 100 nM (0.0747 ng/cm2) and 500 nM (0.374 ng/cm2) Fl was nebulized 24 h after PHMG treatment.

All untreated cells were nebulized only with the vehicle control (0.9% NaCl solution). Control (CTRL) samples were maintained within the same AX12 plate (for example, Chip A nebulized with control solvent and chip B with test substance) to remove bias effects of handling.

### qRT-PCR

Total RNA was isolated separately from apical and basal chamber and subsequently purified using the Direct-zol™ RNA Microprep kit (Zymo Research, Switzerland) using the manufacturer protocol. Purity and concentration of RNA was analyzed using a Nano-Drop Spectrophotometer (Thermo Fischer Scientific, Switzerland). Next, cDNA was transcribed using the Super Script III Reverse Transcriptase kit (Life Technologies, Switzerland). Finally, qRT-PCR reactions were performed in triplicates with SYBR^®^ Select Master Mix (Thermo Scientific) in an ABI7500 Fast (Applied Biosystems) real-time PCR system. Target gene expression was normalized to housekeeping gene expression (HPRT). The primer sequences are provided in Supplementary Table S1.

### Immunofluorescence staining and imaging

Cells on-chip were fixed using 4% paraformaldehyde in distilled PBS- (PBS without calcium and magnesium). “Medium exchange” function on the AX Exchanger was used to fix the cells within the basal chamber. Chips were unscrewed and opened using the ^AX^Disassembly tool (AlveoliX, Switzerland) prior to staining and mounting.

Cells were first permeabilized using 0.1% Triton X-100 (Sigma-Aldrich, Germany) dissolved in PBS- for 15 min at RT and then blocking buffer (2% BSA in sterile PBS) was added. The cells were incubated for 1 h at RT with the blocking buffer. The primary antibodies used for the study are mouse anti-ZO-1 antibody (1:100 dilution; C# 33-9100, Fisher T Scientific), rabbit anti-α-SMA antibody (1:200 dilution; #SAB5500002; Sigma-Aldrich). The primary antibodies were diluted in 2% BSA/PBS and incubated overnight at 4°C. Secondary antibodies were used as follows: donkey anti-mouse Alexa Fluor 488 (1:200 dilution; C# A21202, Invitrogen), donkey anti-rabbit Alexa Fluor 568 (1:200 dilution; C# A10042, Invitrogen), were diluted 1:2000 in 2% BSA/PBS and incubated 2 h at RT. Nuclei were stained with Hoechst (1:1000 dilution; 33342, Invitrogen). The actin cytoskeleton was visualized using the conjugated Alexa Fluor 647 phalloidin stain (1:300 dilution; C# PHDN1-A, Cytoskeleton, Inc.). Lastly, the stained membranes were sealed between two glass coverslips using a mounting medium (C# F6182, Sigma-Aldrich).

Images were obtained using a confocal laser scanning microscope (Zeiss LSM 710) using appropriate filter settings. Zen Blue software v2.1 (Zeiss) was used for fluorescent intensity calculation to obtain background-corrected mean fluorescence intensity (MFI) for each channel of interest. To obtain comparable results from different areas of interest on the membrane, identical settings for the optical and digital gain, area of focus, and laser intensity were maintained. Finally, the mean fluorescence intensity (MFI) of the test channel (red, αSMA and for Phalloidin) was normalized with the MFI of the nuclei channel in blue (stained with Hoechst).

### LDH cytotoxicity assay

Collected supernatants from the cells on-chip were analyzed for Lactate Dehydrogenase (LDH) release. The LDH cytotoxicity detection kit (Roche, #1164479301) was used according to the manufacturer’s protocol. Briefly, for each sample equal volumes of supernatant to LDH reaction buffer was prepared and then transferred into 96-well plates in triplicate. The respective cell culture medium was taken as the “blank” control. Healthy untreated cells were considered as “negative” control for the assay, whereas cells treated with 1% Triton X-100 for 4 h were considered as the “positive” control for maximum LDH release. 30 min after incubation with the LDH reaction mixture at RT, absorbance at 490 nm and a reference wavelength at 600 nm was measured using a microplate reader (Tecan Reader M1000).

### Cellular ROS assay

To measure reactive oxygen species (ROS) from NPs exposed cells, the DCFDA/H2DCFDA cellular ROS assay kit (Abcam; ab113851) was used according to the manufacturer’s instructions. In summary, the cell culture medium was aspirated out from the wells and the cells were washed with 1X Buffer (provided in the kit). Next, 100 μL of DCFDA solution was added to each well and subsequently incubated for 4 h protected from light at RT. The DCFDA solution was then carefully aspirated out and replaced with 100 μL 1X Buffer. A blank or negative control (well with no cells) was included to normalize the background for subtraction. A positive maximal ROS control was prepared with Ter-butyl hydrogen peroxide (TBHP) at a concentration of 150 μM in 1X buffer. DCF on-chip was then finally detected at Ex/Em = 485/535 nm in a fluorescence microplate reader (Tecan Reader M1000).

### Statistical analysis

All data are presented as mean ± standard error of the mean (SEM) unless specified otherwise. For AX12 plate experiments, “N” represents the experimental repetitions, and “n” represents the number of individual wells estimated for all experiments with mRNA and cell supernatants, two wells were pooled into 1 and referred to as n = 1. All statistical tests were performed using t-tests, one-way or two-way analysis of variance (ANOVA) with *p* values adjusted for multiple comparisons using Tukey’s multiple comparisons test, *p < 0.0332, **p < 0.0021, ***p < 0.0002, ****p < 0.0001. GraphPad Prism v8.0 software was used for data analysis. The exact number of repeats performed for each experiment is indicated in the corresponding figure legends.

## Results

### The cloud α AX12 system

To estimate the QCM 6 precision in the Cloud α AX12, theoretically calculated mass deposition (using Eq. 2, in *Methods*) was compared with the measured QCM 6 signals (actual mass deposited) for fluorescein concentrations. Here an average difference of 15% was found, which indicated that the actual mass deposition determined from the QCM 6 signal was 85% (SD: 5%) of the theoretically estimated mass deposition (Figure 2C). Thereafter, the spatially uniform and homogeneous dose distribution in the wells of the AX12 plate were assessed using a fluorescein concentration of 15 μg/mL.

The nebulization volume and the choice of base liquid have a major effect on the toxicant deposition and on their distribution in the exposure chamber. Hence, we tested two SBL, namely, 1% PBS in distilled water (PBS) and 0.9% NaCl in distilled water (NaCl) with nebulization volume ranging from 100 to 500 µL. The output rate (O.R.) of the nebulizer for the SBL is a key parameter, ideally staying between 0.2 and 0.4 mL/min, ensuring uniform cloud formation, which is essential for the homogeneity of aerosol deposition. The O. R is significantly affected by the choice of SBL used and the solubility of the test compounds within the SBL. It is calculated by measuring the time needed for the liquid (volume nebulized) to pass through the mesh nebulizer. Our results confirmed that there were no significant differences in the O.R. of the two SBLs used (Figure 2D). Hence, 0.9% NaCl was chosen as the SBL for all the consecutive studies (Table 1). Consistency of O.R. was tested by using different solutions are mixed with test compounds, PHMG was mixed with NaCl solution. A drastic reduction from 0.4 (NaCl only) to 0.2 mL/min (NaCl with PHMG) in O.R. was observed (Figure 2E), which was expected due to the increased viscosity of the liquid suspension used for nebulization. However, the values were within the manufacturer recommended range. Lastly, the fluorescence intensities of nebulized fluorescein measured by a plate reader showed a variation of ±8.5% fluorescein deposition within the AX12 plate (Figure 2F), which is within an acceptable range (up to 15%) according to the manufacturer (Vitrocell).

## Nanoparticles (NPs)-induced inflammation on-chip

### Cyclic stretch combined with ALI increased sensitivity of lung epithelial barrier to NPs

**FIGURE 3:**
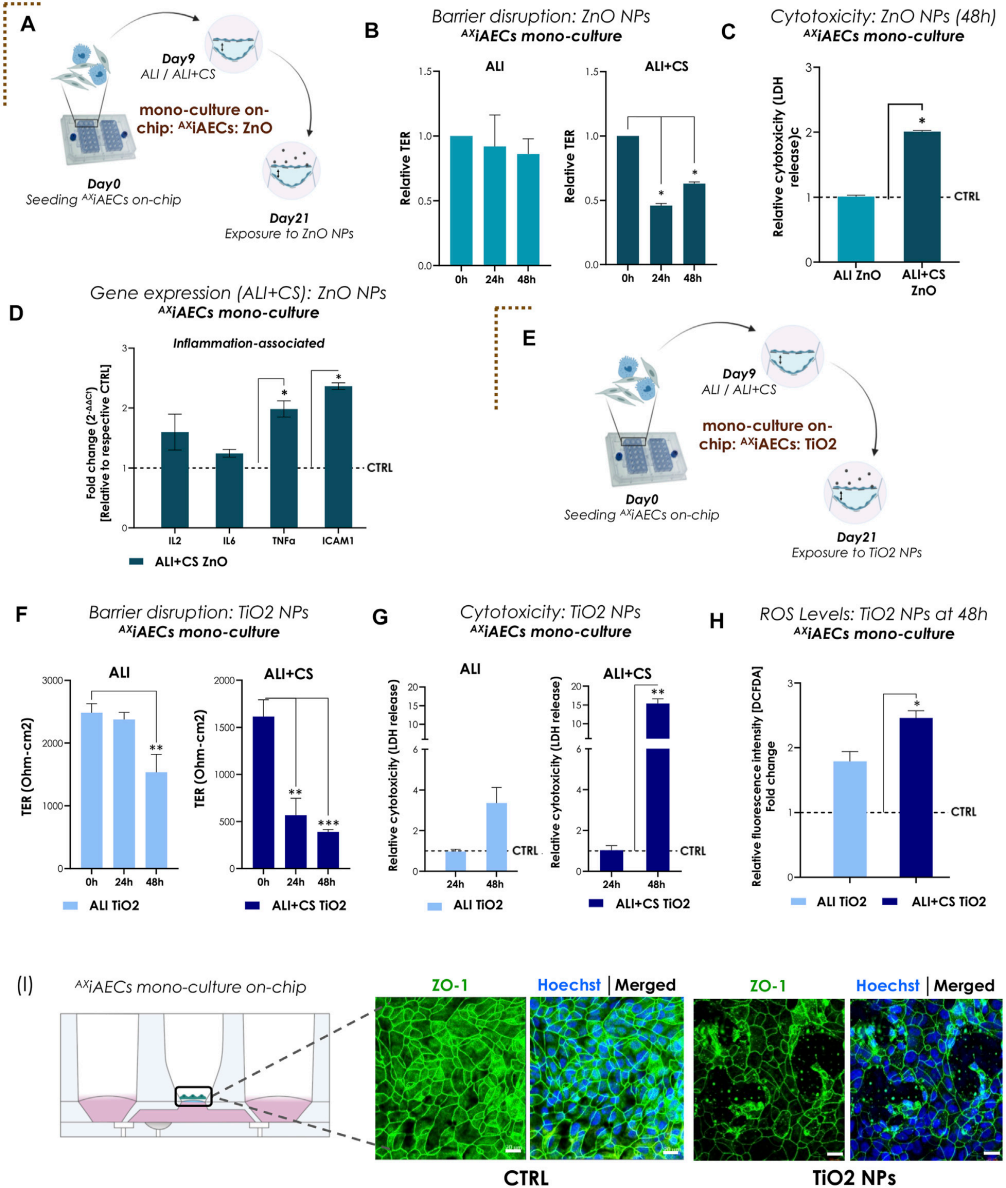
Nanoparticles (NPs)-induced inflammation and barrier disruption in ^AX^iAECs mono-culture on-chip **(A)** The brown dotted corner bracket suggests the distinction between different trigger-induced models. On day 21 of the mono culture experiment, the ^AX^iAECs were exposed to the ZnO NPs on-chip. Timeline and schematic provide further details of the experiment. **(B)** TER values (normalized to 0 h timepoint) for ALI and ALI + CS cell cultures exposed to ZnO NPs (n = 3/condition/timepoint). **(C)** Relative LDH release (NPs exposed with respect to Control; CTRL) shown for 48 h timepoint for ALI and ALI + CS cells (n = 4). **(D)** mRNA was isolated from CTRL and ZnO exposed cells (ALI + CS) at 48 h exposure timepoint. qPCR studies were performed with n = 4/conditions and exposure significance were measured in relation to CTRL expression levels. **(E)** Results for ^AX^iAECs mono-cell culture model with TiO2 NPs is denoted by opening another brown dotted corner bracket. Timeline and schematic of TiO2 NPs nebulization on day 21 in monoculture (^AX^iAECs) on-chip. **(F)** Absolute TER values (Ohm-cm2) compared pre-TiO2 NPs exposure (at 0 h) with 24h and 48 h after exposure (N = 2; n = 6). **(G)** Significance for cytotoxicity was calculated relative to respective ALI or ALI+CS CTRL. Cytotoxicity was calculated from LDH release at 24h and 48 h after TiO2 NPs nebulization from both ALI and ALI + CS samples (N = 2; n = 4/time-point). **(H)** ROS generation was measured using the H2-DCFDA assay (N = 2; n = 4). Fluorescence intensity for exposed cells were normalized with respective healthy CTRL. **(I)** Representative immunofluorescence staining for alveolar barrier after 48 h of TiO2 NPs exposure. Cells were probed for tight junction, ZO1 (green) and nuclei with Hoechst (blue). Scale bar is 20 µm. Data are shown as mean ± SEM.

For the first proof-of-concept study mimicking physiologically relevant environmental cues, TiO2 and ZnO NPs were used. We investigated the inflammatory potential of ZnO NPs (0.21 μg/cm^2^) on bronchial (Calu3; Supplementary Figure S2A) and alveolar (^AX^iAECs; Figure 3A) mono-cell culture on-chip. One of the most critical parameters for toxicity studies *in vitro* is the existence of a tight and functional lung epithelial barrier (Derk et al., 2015). Hence, the barrier integrity for Calu3 cells cultured on-chip in healthy conditions was investigated and found to be around 1000 to 3000 Ω-cm2 between Day D)7–14 under both ALI and ALI + CS conditions (Supplementary Figure S2B). Barrier disruption was assessed before (at 0 h) exposure and 6 h and 24 h after nebulization with ZnO NPs for Calu3 cells on-chip. We found that aerosolized ZnO NPs exerted significant barrier gaps in Calu3 cells (Supplementary Figure S2C) reducing the TER values 3-folds at 6 h and around 7-folds at 24 h after exposure under ALI + CS conditions. Furthermore, significant increase in cytotoxicity levels was observed under ALI + CS conditions at 24 h (Supplementary Figure S2D). ^AX^iAECs monoculture on-chip showed similar barrier disruption upon exposure to ZnO NPs at 24 h (45% reduction) and 48 h (62% reduction) but only under ALI + CS conditions (Figure 3B). In addition, LDH release was found to be almost 2-times higher under ALI + CS condition than under ALI condition alone in ^AX^iAECs on-chip (Figure 3C). qPCR studies (measured in Fold change; fc) revealed increased levels of inflammation-associated genes notably interleukin 2 (IL2; fc in Calu3 21.46; ^AX^iAECs 1.60) and TNFα (fc in Calu3 17.98; ^AX^iAECs 1.98) among others in both ^AX^iAECs and Calu3 cells on-chip under ALI + CS conditions when exposed to ZnO NPs (Figures 3D, Supplementary Figure S2E). In favor of an active inflammation, reduced expression of epithelial associated genes such as aquaporin 5 (AQP5), caveolin 1 (CAV1) in ^AX^iAECs (Supplementary Figure S2G) and tight junction protein 1 (TJP1) and e-cadherin (ECAD) were observed in Calu3 cells (Supplementary Figure S2F) confirming a strong effect on their epithelial characteristics.

Next, ^AX^iAECs on-chip were nebulized apically with TiO2 NPs (Figure 3E). Exposure to nebulized TiO2 NPs (0.21 μg/cm^2^) demonstrated significant barrier damage in ^AX^iAECs as revealed from their TER values after 48 h under ALI (38% decrease) and from 24 h under ALI + CS (24 h: 64%; 48 h: 75%) conditions (Figure 3F). Consistent with this, immunofluorescent stainings of ZO1 revealed substantial tight junction disruptions (Figure 3I) along with reduced proportion of actin filaments compared with healthy control cells (Supplementary Figure S1C). Nebulized high concentration of TiO2 NPs (0.21 μg/cm^2^) showed increased cytotoxicity (15.3 folds) under ALI + CS cells from 48 h (Figure 3G), whereas low concentration of TiO2 NPs (0.021 μg/cm^2^; 10-fold diluted) were unable to demonstrate any cytotoxic effect (Supplementary Figure S1D) either under ALI or ALI + CS conditions. Previous studies have shown that TiO2 NPs are a known trigger for the generation of reactive oxygen species (ROS) in lung cells and this increased ROS production plays a direct role in lung inflammation (Sayes et al., 2006; Horváth et al., 2018). Consistent with published literature, a high concentration of TiO2 NPs (0.21 μg/cm^2^) nebulized with the Cloud α AX12 significantly increased ROS production in the alveolar cells after 48 h under ALI + CS conditions (Figure 3H). Further qPCR studies in ^AX^iAECs revealed deregulation of inflammatory genes like interleukin 6 (IL6; fc: for ALI 1.13; ALI + CS 1.85), tumor necrosis factor α (TNFα; fc: for ALI 1.03; ALI + CS 2.25) and intercellular adhesion molecule 1 (ICAM1; fc: for ALI 0.95; ALI + CS 2.86) which displayed a strong trend towards increased activation when exposed to high concentration of TiO2 NPs (Supplementary Figure S1E).

In our alveolar and bronchial mono-cell culture model on-chip, ALI combined with CS conditions showed increased barrier sensitivity and thus increased inflammatory potential of NPs nebulized with the Cloud α AX12.

### Higher inflammatory potential of TiO2 NPs under ALI + CS established using a triple-cell culture model on-chip

**FIGURE 4:**
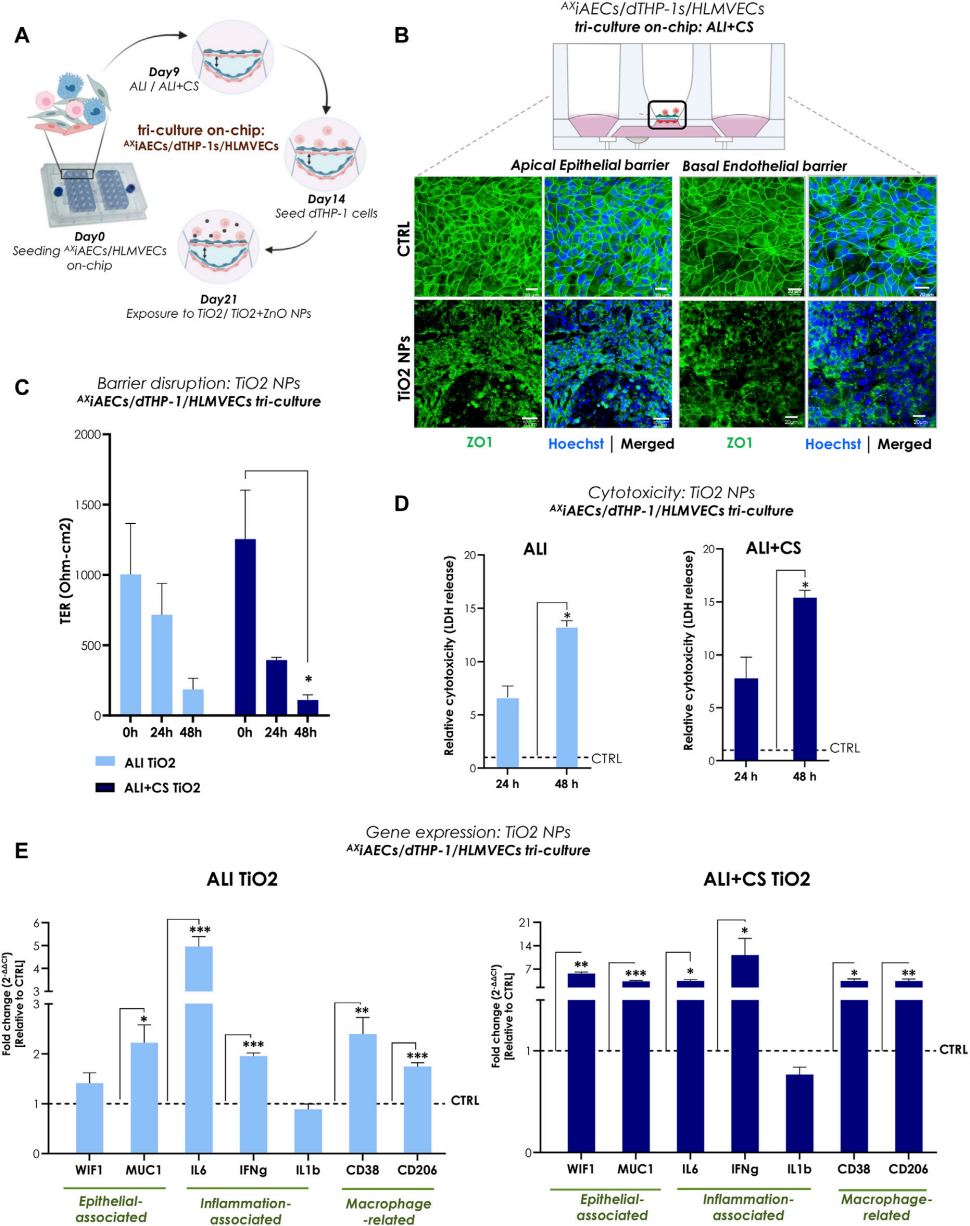
TiO2 NPs-induced inflammation and barrier disruption in triple-cell culture model on-chip **(A)** Overview of the timeline for cell seeding and NPs exposure on-chip. **(B)** Representative immunofluorescent (maximum projection intensity) images for alveolar apical and basal endothelial barrier stained with ZO1 (in green) and nuclei with Hoechst (in blue) under ALI + CS. Scale bar here is 20 µm. **(C)** TER (Ohm-cm2) measured before at 0 h and after TiO2 NPs exposure at 24 h and 48 h (N = 2; n = 6). **(D)** LDH release was measured from 24 h to 48 h samples in ALI and ALI + CS conditions (N = 2; n = 4/conditions/time-point). Significance was measured relative to the respective ALI or ALI+CS CTRL. **(E)** mRNA was harvested at 48 h time-point. Gene expression for epithelial, inflammation and macrophage related markers were measured in both ALI and ALI + CS conditions (N = 2, n = 4/conditions). Data are shown as mean ± SEM.

To reproduce a complex and physiologically relevant *in vitro* model of the alveolar-capillary barrier, a triple (tri)-cell culture was established on-chip with ^AX^iAECs and differentiated THP1 (d-THP1) macrophages on the apical side of the cell-seeding membrane and HLMVEC endothelial cells on the basal side under ALI (Supplementary Figure S3A, B) and ALI + CS (Figures 4A, B). Interestingly, both the apically exposed alveolar cells and the basal indirectly exposed endothelial barriers revealed disrupted tight junction proteins when exposed to TiO2 NPs under ALI + CS state (Figure 4B). TER measurements before (0 h) and after exposure revealed significant decrease in barrier health at 48 h (80% reduction) under ALI + CS conditions (Figure 4C). In line, increased cytotoxicity from 48 h under both ALI (43% cytotoxicity) and ALI + CS (59% cytotoxicity) conditions (Figure 4D) was observed. Comprehensive gene expression analysis revealed activation of pro-inflammatory markers like IL6 (fc under ALI 4.95; ALI + CS 3.47) and interferon gamma (IFNg; fc under ALI 1.96; ALI + CS 11.14) under both ALI and ALI + CS states. Moreover, increased expression of Mucin1 (MUC; fc under ALI 2.21; ALI + CS 3.34) and Wnt inhibitory factor 1 (WIF1; fc under ALI + CS 5.65) along with deregulated levels of CD38 (fc under ALI 2.39; ALI + CS 3.47) and CD206 (fc under ALI 1.74; ALI + CS 3.41) suggest epithelial and macrophage activation respectively (Figure 4E).

In summary, exposure to TiO2 NPs (approximate *in vivo* dose correlation, see Supplementary Section S1) resulted in significantly reduced alveolar barrier integrity along with inflammation and cellular damage.

### Nebulized NPs individual or in combination reproduced barrier gaps and inflammation in primary human alveolar cells on-chip

**FIGURE 5:**
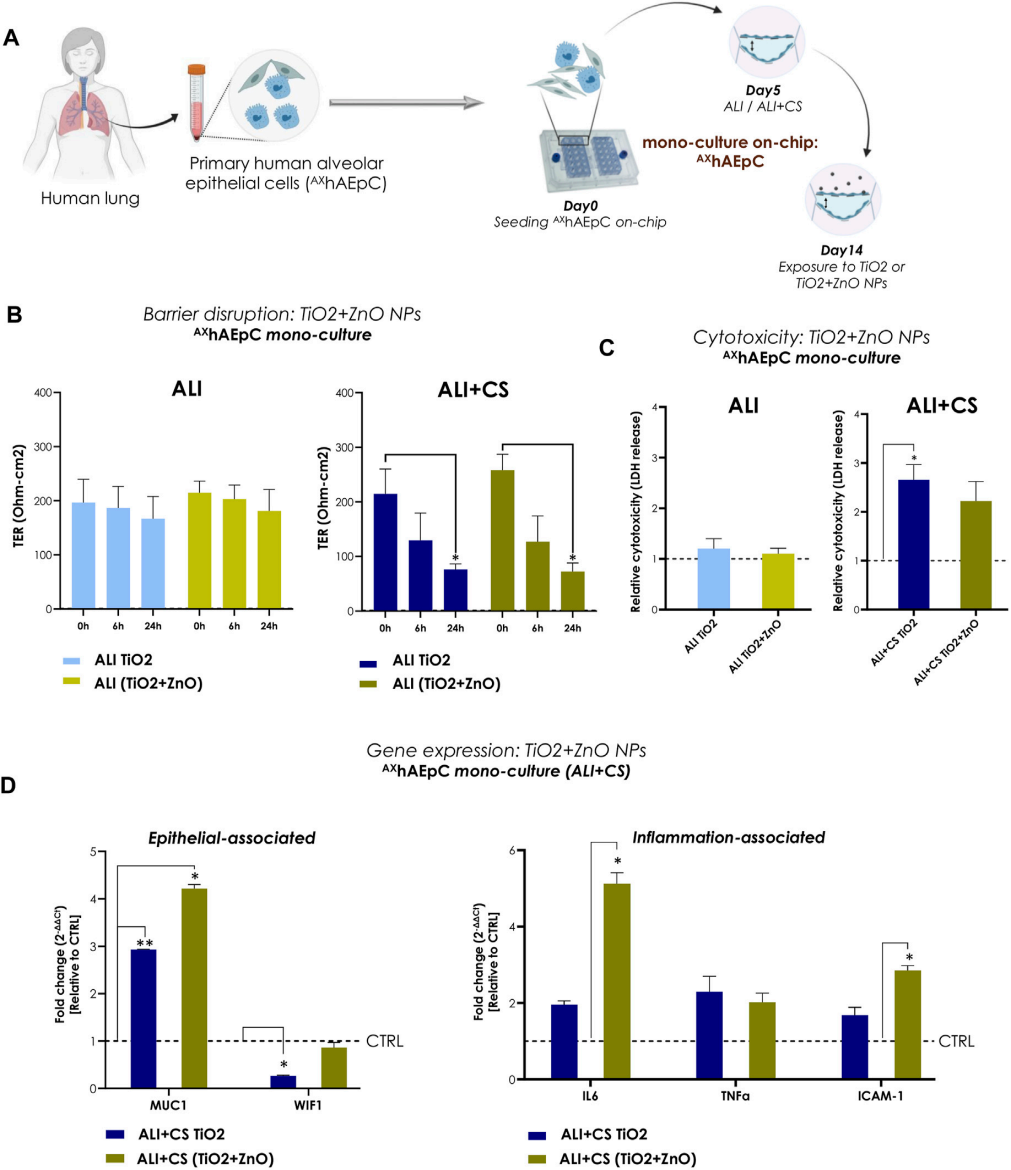
NPs-induced inflammation in primary human alveolar epithelial cells (^AX^hAEpC) on-chip **(A)** A schematic representation ^AX^hAEpCs seeded in the AX12 plate. The timeline shows the cell culture and NP exposure process on-chip. **(B)** TER values (Ohm-cm2) were recorded before NPs nebulization (at 0 h) and at 6h and 24 h after TiO2 and TiO2+ZnO mixed NPs exposure (N = 2; n = 6). The significance of exposure at 6h and 24 h was measured compared with the values at 0 h. **(C)** The levels of LDH at 24 h were normalized using untreated control samples (ALI and ALI + CS) (N = 2, n = 4). **(D)** Gene expression was analyzed 24 h after NPs exposure using mRNA harvested from ALI + CS cell cultures. The fold change values were normalized to healthy controls (N = 2, n = 4). Data are shown as mean ± SEM.

^AX^iAECs/dTHP-1/HLMVECs tri-cell culture on-chip were additionally exposed to mixed TiO2+ZnO NPs combination. Here, the NPs mixture were able to incite strong barrier disruption after 24 h (64.5% reduction) and 48 h (90.1% reduction) in ALI + CS (Supplementary Figure S2H) and elevated levels of inflammatory genes like interleukin 1b (IL1b; fc in ALI 2.62; in ALI + CS 3.60) and IFNg (fc in ALI 2.22; in ALI + CS 4.42) were observed under both CS and non-CS (ALI only) conditions (Supplementary Figure S2I).

To recapitulate realistic occupational exposure of NPs in industrial spaces, we used equal parts of aerosolized TiO2 (0.10 μg/cm^2^) and ZnO NPs (0.10 μg/cm^2^) to test effect of NP combination. Primary human alveolar epithelial cells (^AX^hAEpC) consisting of a pure AT2 cell population are still considered the “gold standard” for use as a distal lung cell model (Elbert et al., 1999). Therefore, to validate the Cloud α AX12, we utilized ^AX^hAEpCs (Figure 5A). ^AX^hAEpCs on-chip when exposed to nebulized TiO2 NPs alone and in combination with ZnO NPs demonstrated significant increase in barrier gaps under ALI + CS condition, as evidenced by reduced TER values at 6 h (TiO2: 67.4% decrease; TiO2+ZnO: 46.4% decrease) and 24 h (TiO2: 73.7% decrease; TiO2+ZnO: 69.1% decrease) after induction (Figure 5B). While no cytotoxic effects were observed in ALI conditions, significantly increased LDH release was seen under ALI + CS condition, with cells exposed to TiO2 NPs (Figure 5C). RNA was harvested from ALI + CS cells after 24 h exposure NPs exposure. Subsequent qPCR studies revealed deregulated epithelial-associated genes such as MUC1 (fc for TiO2 2.93; for TiO2+ZnO 4.21) and WIF1 (fc for TiO2 0.26) with increased inflammatory genes like IL6 (fc for TiO2+ZnO 5.1) and ICAM1 (fc for TiO2+ZnO 2.8) (Figure 5D).

Therefore, both primary ^AX^hAEpCs and the cell-line models demonstrated similar patterns of inflammation and toxicity when exposed to TiO2 NPs alone or in combination (TiO2+ZnO NPs) using the Cloud α AX12 reinforcing the validity of our findings with diverse cellular models.

## Toxic chemical “inhalation” on-chip

### Nebulized PHMG induced cytotoxicity in alveolar triple-cell culture model on-chip

**FIGURE 6:**
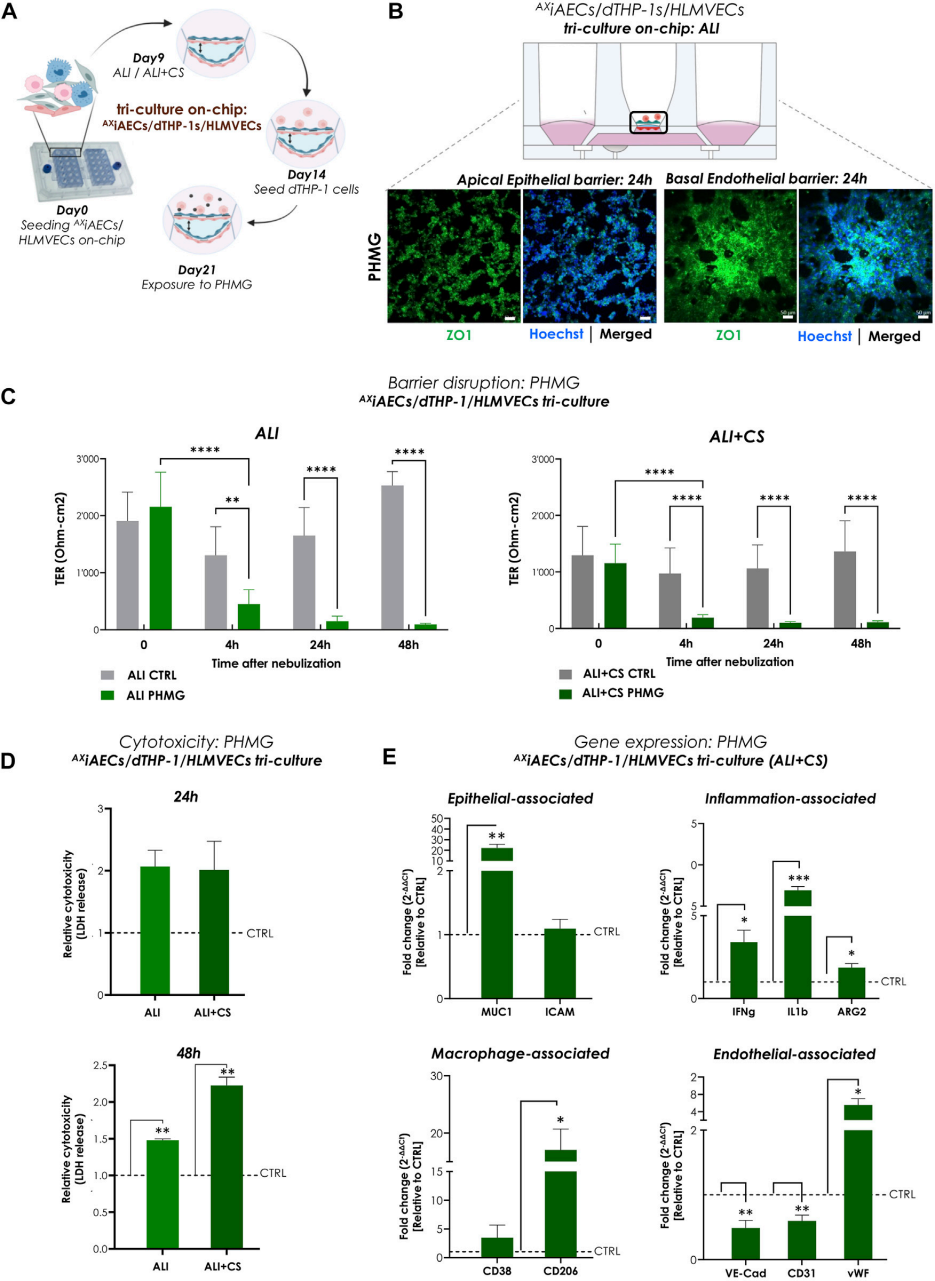
Aerosolized PHMG induced barrier disruption and cytotoxicity in triple-cell culture on-chip **(A)** Timeline and schematic of PHMG nebulization at day 21 in tri-cell culture (^AX^iAECs/d-THP1/HLMVEC) model on-chip. **(B)** Representative immunofluorescence staining for apical alveolar and basal endothelial barrier after 24 h of PHMG exposure. Cells were analyzed for tight junction, ZO1 (green) and nuclei with Hoechst (blue). Scale bar is 50 µm. **(C)** TER values (Ohm-cm^2^) were recorded before PHMG nebulization (at 0 h) and at 4 h, 24 h and 48 h after exposure (N = 3; n = 12). **(D)** Cytotoxicity was calculated from LDH release at 24 h and 48 h after PHMG nebulization from both ALI and ALI + CS conditions. LDH release was normalized with respective untreated controls. Data shown as mean ± SEM (N = 1; n = 3/condition/timepoint). **(E)** Gene expression after 48 h of exposure was assessed for cells in ALI + CS condition. Fold change values were normalized with respective untreated controls. Data presented as mean ± SEM (N = 3; n = 6).

To investigate the effect of aerosolized PHMG, a humidifier disinfectant (Park J. et al., 2019), on a cellular level, both ^AX^iAECs/d-THP1s co-culture and ^AX^iAECs/d-THP1s/HLMVEC tri-cell culture model was utilized (Figures 6A, Supplementary Figure S5A). A significant reduction in TER (ohm-cm2) measurements was observed in the tri-cell culture starting as early as 4 h after nebulization and remained constant until 48 h under both ALI and ALI + CS cultured cells (Figure 6C). However, in the ^AX^iAECs/d-THP1s co-culture model, barrier disruption was observed only at 24 h but remained constant until 48 h (Supplementary Figure S5B). In addition, Calu3 cells on-chip showed decreased barrier integrity from 24 h when exposed to PHMG under ALI + CS (Supplementary Figure S4B). The presence of epithelial cells and macrophages on the apical side along with basal ECs was assessed with Imaris. CD68^+^ expression confirmed the presence of differentiated THP1 (d-THP1) macrophages on the apical side (Supplementary Figure S3A, B). Consistent with the TER measurements, immunofluorescent stainings revealed drastic disruption of tight junction in both the apical alveolar and basal endothelial barriers under ALI (Figure 6B) and ALI + CS (Supplementary Figure S4A) in the tri-cell culture model. Moreover, increased CD163+ macrophages were observed upon PHMG exposure (Supplementary Figure S4A) under ALI + CS condition, confirming an ongoing inflammatory response (Skytthe et al., 2020). Furthermore, substantial LDH release was observed after 48 h under ALI and ALI + CS conditions the tri-cell culture (Figure 6D) confirming the cytotoxicity of PHMG (Song et al., 2019). Investigation of the gene expression profile after PHMG exposure in the co-culture revealed a significant increase in inflammatory markers such as arginase 2 (ARG2; fc: 1.81) and an increase M1-associated marker such as CD38 (fc: 1.76) (Supplementary Figure S5C). qPCR studies from the tri-cell culture, under ALI + CS cells revealed elevated levels of inflammatory genes like IFNg (fc: 3.39), IL1B (fc: 6.92) and ARG2 (fc: 1.86). In addition, the epithelial damage-related marker MUC1 (fc: 22.17) was found to be significantly elevated. Both M1-and M2-associated markers like CD38 (fc: 3.46) and CD206 (fc: 17.03) respectively showed increased levels upon PHMG induction. On the basal side, gene expression studies of endothelial cells identified decreased mRNA levels of VE-CAD (fc: 0.49) and Platelet endothelial cell adhesion molecule-1 (PECAM-1 or CD31; fc: 0.59), but increased levels of von Willebrand factor (vWF; fc: 5.54) (Figure 6E).

In summary, PHMG exposure in mono-culture, co-culture and tri-cell culture models on-chip resulted in drastic barrier disruption, increased cytotoxicity along with significant inflammation and cellular differentiation.

### Aerosolized fluticasone (FL) corticosteroid reduces PHMG-induced EMT and inflammation

**FIGURE 7:**
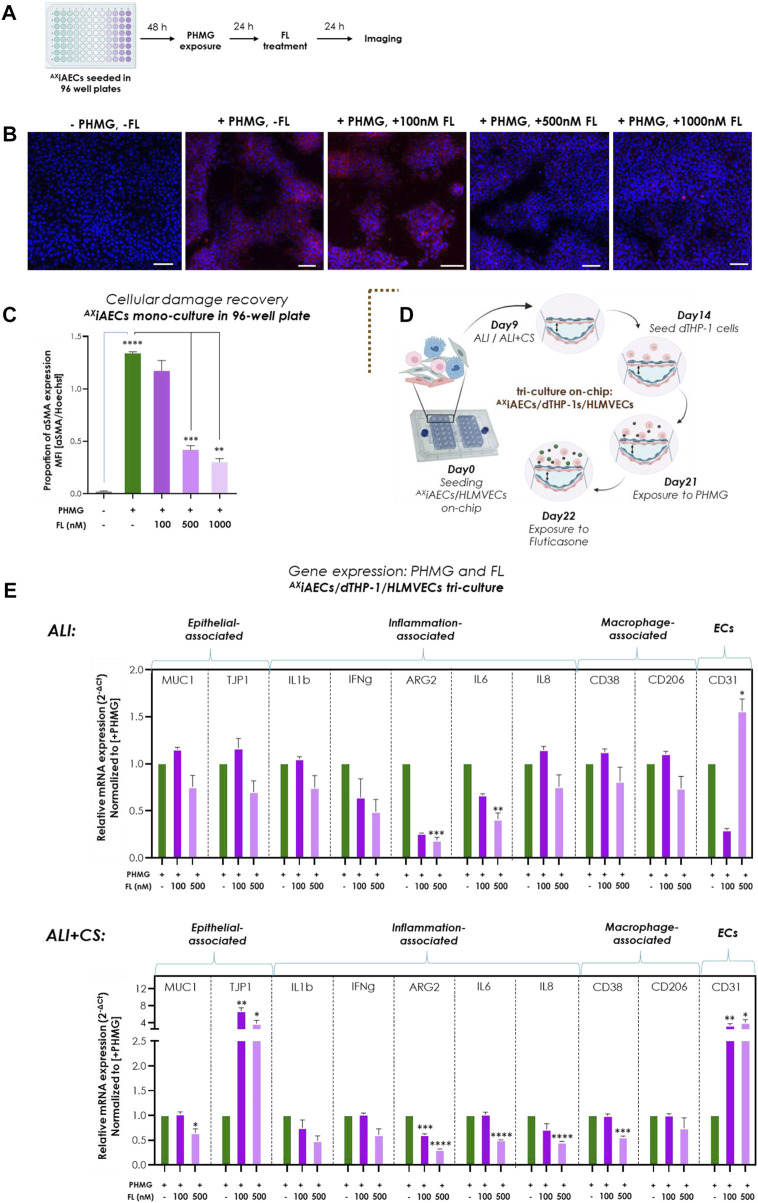
Inhaled Fluticasone reduced PHMG-induced inflammation and EMT **(A)** Schematic timeline of the PHMG exposure and subsequent FL treatment on ^AX^iAECs mono-culture in 96- well plates. **(B)** Representative stainings of ^AX^iAECs exposed to PHMG with/without FL treatment under ALI with αMA (in red) and Hoechst (in blue). Scale bar is 100 µm. **(C)** The mean fluorescent intensity (MFI) was calculated and the proprotion of αSMA expression was calculated by normalizing with the nuclei intensity. PHMG exposed cells (+PHMG, -FL) was compared with healthy untreated controls (-PHMG, -FL) to check inflammation by PHMG. Next, PHMG exposed cells (+PHMG, -FL) were compared with PHMG and FL treated cells (+PHMG, +100nM/500nM/1000 nM FL) to check anti-inflammatory effect of FL. Region of interest (ROI or n = 6/conditions). Data are shown as mean ± SEM. **(D)** Brown dotted brackets represent on-chip experiments. Timeline and schematic for PHMG and FL treatment in tri-cell culture (^AX^iAECs/d-THP1s/HLMVECs) on-chip. **(E)** Selected genes for epithelial cells, inflammation-associated, macrophage and endothelial cells were measured under both ALI and ALI + CS conditions on-chip. Significance associated with FL treated cells were estimated by comparing inflamed cells (+PHMG, -FL) with 100 nM (+PHMG, +100 nM FL) and 500 nM (+PHMG, +500 nM FL) FL nebulized cells (N = 2, n = 8). Data are shown as mean ± SEM normalized to PHMG induced but without subsequent FL (+PHMG -FL) treatment.

To evaluate the applicability of the Cloud α AX12 as a setup for testing the efficacy of inhaled drugs, we used the corticosteroid FL subsequently with the PHMG-induction on-chip. Initial testing was performed using 96- well plates in which FL was nebulized 24 h after PHMG induction on ^AX^iAECs mono-culture (Figure 7A). Since, PHMG is known to induce epithelial-mesenchymal transition (EMT) in alveolar cells (Park Y. et al., 2019), protein expression of alpha smooth muscle actin (αSMA) as a mesenchymal cell marker was used as an indicator of EMT level. While PHMG exposure significantly increased αSMA levels, as expected, inhaled treatment with FL showed a dose-dependent reduction in αSMA protein expression combined with an improvement in cellular health (Figures 7B, C). To test this hypothesis on-chip, the ^AX^iAECs/d-THP1s/HLMVEC tri-cell culture model was used. The FL treatment was carried out 24 h after PHMG exposure on day 22 (Figures 7D, Supplementary Figure S6A). FL exposure (Supplementary Figure S6A) was unable to recover the strong barrier damage inflicted by PHMG under both ALI (Supplementary Figure S6B) or ALI + CS (Supplementary Figure S6C) conditions. However, detailed qPCR studies indicated clear effects of the FL treatment under ALI + CS conditions. mRNA was isolated from cells, 24 h after FL treatment under ALI and ALI + CS conditions. Alveolar (TJP1) and endothelial (CD31) barrier-associated markers were increased after FL treatment, while the expression of alveolar damage marker (MUC1) and the inflammatory (ARG2, IL6 and IL8) gene levels (Figure 7E) were reduced. Moreover, treatment with aerosolized FL was able to significantly reduce the cytotoxic effect (55% decrease with 100 nM and 78% decrease with 500 nM) of PHMG under ALI + CS cells in a dose-dependent manner (Supplementary Figure S6D).

Briefly, the nebulized corticosteroid FL (approximate *in vivo* dose correlation, see Supplementary Section S1) provided significant anti-inflammatory effects that could be shown with the Cloud α AX12, demonstrating its potential for use in drug efficacy studies.

## Discussion

We are constantly being exposed to a broad range of unidentified toxicants. Substances capable of penetrating deep into the distal part of the lungs leading to severe respiratory conditions such as ARDS, COPD and fibrosis (Adegunsoye & Rafeq, 2019). Due to the increasing incidence of these respiratory diseases, there is a growing demand for new inhaled targeted therapeutics for the lung. It is therefore of utmost importance to understand the response of the lung epithelial barrier to inhaled toxicants to develop effective treatments for such debilitating health conditions. Accordingly, we present here a new generation *in vitro* inhalation platform (Cloud α AX12) for the distal lung consisting of a cloud-based exposure chamber combined with a lung-on-chip technology (^AX^Lung-on-chip). Exposure to aerosolized NPs (like TiO2, ZnO) and chemical (like PHMG) was shown to trigger an inflammation insult leading to epithelial barrier disruption and cytotoxicity on-chip. Our study reveals a clear effect of physiological levels of CS associated with ALI cell culture. In addition, the anti-inflammatory potential of inhaled FL treatment with the Cloud α AX12 platform, was demonstrated showing its applicability for aerosolized drug screening studies.

Although air-liquid exposure systems better model *in vivo* scenarios compared to instilled compound testing strategies, the interpretation and correlation with *in vivo* dosing remains a major challenge. Nevertheless, the integration of accurate QCM 6 in exposure systems for ALI culture studies have improved the accuracy and reduced the variability of the dose deposition. In our studies, we observed a ∼8.5% variability which is consistent and in agreement with previous studies using earlier generations of the Vitrocell Cloud devices (Lenz et al., 2014; Ding et al., 2020). In contrast to earlier studies using ZnO and TiO2 NPs to study lung barrier toxicity (Y. H. Kim et al., 2010; Oliveira et al., 2019) carried out with concentrations ranging from 0.4 to 0.9 μg/cm^2^ without CS inclusion (Ding et al., 2020), we observed significant cytotoxicity at even lower NPs concentrations (0.21 μg/cm^2^) with nebulized TiO2 and ZnO NPs under ALI + CS. This suggests an effective aerosol to cell delivery of the Cloud α AX12 and the impact of physiological CS on the uptake of NPs. Moreover, an inter-laboratory study conducted in collaboration with 7 different laboratory groups recently confirmed the homogenous dispersion of nanoparticles and consistent dose deposition profile using the VITROCELL^®^ Cloud 12 system (Bannuscher et al., 2022). This study is a significant advancement in the standardization of *in vitro* toxicity studies using ALI culture conditions and advanced cloud exposure devices for regulatory purposes.

Although, there is still much debate and a lack of standardized protocols for correlating *in vivo* and *in vitro* dosing, especially for inhaled agents, our basic worst-case lifetime calculations indicate that the 100 μg/mL dose used in our studies correlate approximately with 18–60 h exposure for ZnO NPs and a 45–90 h exposure for TiO2 NPs for an adult human living in a highly polluted area (Supplementary Section S1). The difference in exposure time for ZnO and TiO2 is related to their differences in solubility and hydrophobicity profile (TiO2 is more hydrophobic than ZnO) which determine their deposition kinetics in the lung (J. Wang & Fan, 2014; IARC, 1989). The lack of standardized protocols may of course also explain the difference observed. It would therefore be important to understand the role of surface modifications of NPs and how that influence their uptake mechanism in the lungs. PHMG was chosen as a model for chemically induced toxicity because recent cases of severe lung toxicity and inflammation in Korea led to permanently ban PHMG as a component in humidifier disinfectants (Park D. et al., 2015). In addition, several compelling studies have shown that PHMG is a causative agent for lung toxicity, asthma, fibrosis and cancer using *in vivo* animal inhalation studies (S. R. Kim et al., 2015; S. H; Lee et al., 2021; Y. H; Lee & Seo, 2020). *In vitro* studies have shown increased cytotoxicity, ROS generation with EMT (S. Choi et al., 2022; Jeong et al., 2019; Park J. et al., 2019; Shin et al., 2018), which were confirmed on-chip in the present study.

To assess the applicability of the Cloud α AX12 model for simulating inflammation in the upper airway, we included bronchial Calu3 cells in our study. Calu3 cells are commonly used as a cellular model for the upper airway and have been previously shown to exhibit barrier disruption and decreased permeability when exposed to NPs (Banga et al., 2012; Dekali et al., 2014). Our results showed similar barrier disruption in Calu3 cells on-chip when exposed to nebulized NPs (ZnO and TiO2+ZnO NPs; Supplementary Figure S2). Furthermore, gene expression analysis of Calu3 cells exposed to nanoparticles on the chip provides strong evidence of an ongoing inflammatory response (Supplementary Figure S2E). Alveolar ^AX^iAECs were used to model the distal region. Previous studies with those cells have shown lipopolysaccharide (LPS) induced inflammation and EMT mechanism induced by transforming growth factor β 1 (TGFβ1) (Sengupta et al., 2022). We further demonstrate here, that ^AX^iAECs are very sensitive to nebulized NPs and chemicals and show signs of cellular damage, EMT, and a deregulated genetic profile. Furthermore, ^AX^hAEpCs exposed to aerosolized NPs confirmed the results obtained with the ^AX^iAECs model, with a disrupted barrier and a persistent inflammatory insult. Strikingly, increased MUC1 expression on NPs and PHMG trigger was observed in both ^AX^hAEpCs and ^AX^iAECs under dynamic CS conditions. MUC1 is a membrane associated mucin protein that is mainly released by AT2 cells and is present to a lesser extent in AT1 cells (Astarita et al., 2012). Previous research has shown that MUC1 or its partial released fragment, KL6 plays a role in anti-inflammatory reaction specially in diseases such as COPD and cancer (Ishikawa et al., 2011; Milara et al., 2018). Therefore, MUC1 was found in large amount during an active inflammatory phase, reflecting the results of our exposure. WIF1, another extracellular mediator of the Wnt signalling pathway, has recently been associated with clinical relevance in lung inflammation (J. Choi et al., 2020). A sub-population of AT2 cells expressing canonical AT2 markers like surfactant proteins A1, A2 and C (SP-C) among others were found to have increased expression of the WIF1 gene. This AT2 subpopulation and in particular the WIF1 gene have been found to be abundantly expressed during COPD and IPF suggesting their potential role in the pathogenesis of those diseases (Shi et al., 2017; Sauler et al., 2022). This is consistent with our data in which the WIF1 gene expression was increased upon exposure to TiO2 NPs (Figure 4E). This demonstrates the adequate potential of ^AX^iAECs as a cell line model for lung barrier toxicity studies when primary alveolar epithelial cells are not available.

To include immune cells like macrophages in our system as an active player for alveolar inflammation, differentiated THP1 cells were used. d-THP1 cell line is classically used in co-culture studies for the lung to mimic a functional diffusion barrier (Herminghaus et al., 2022). Although, we have used PMA here for differentiation of the THP1 monocytes to activated d-THP1 macrophages according to previously established protocol (Kletting, 2017), several studies have pointed the upregulation of various unrelated genes which overrides the test-inducer associated inflammation (Park E. et al., 2007; Farcal et al., 2012). Future studies are therefore needed to optimize THP1 differentiation protocol to minimize undesirable leaky effects of PMA activation. To verify macrophage activation, CD68 surface protein marker was analyzed after PHMG induction. CD68^+^ macrophages were verified in several studies to be upregulated during inflammation (Chistiakov et al., 2016; Klinge et al., 2020). Hence, increased expression of CD68 surface marker in our studies established the presence of activated macrophage population within the tri-cell culture model (Supplementary Figure S3). Activated CD68^+^ THP1 macrophages were demonstrated to additionally have increased expression of M1-associated macrophage membrane associated protein, CD38 (Lam et al., 2019). Previous research findings have indicated the abundance of CD38 surface marker in THP1 cells during inflammation (Green et al., 2020; Yarbro et al., 2020) which goes in similar direction to what we demonstrated in our model on exposure to NPs (Figure 4E) and PHMG (Figure 7F). While the role of M2-associated macrophage marker CD206 in the lung is not completely established, few studies have shown a correlation of the presence of soluble CD206 forms with acute inflammation cases (Tsuchiya et al., 2019; Nielsen et al., 2020). Since CD206+ M2 macrophage-like cells could be activated through the IL4/IL13 axis (Genin et al., 2015), our hypothesis suggests that exposure to NPs and PHMG trigger an alternative activation of the THP1 cells in co-culture on-chip which might represent an onset for aberrant anti-inflammatory effort. However, further extensive profiling of the inflamed and activated THP1 subpopulations are required to have a conclusive statement.

Furthermore, in our tri-cell culture model, the basal side of the membrane was paved by ECs (HLMVEC). During inflammation, the apical alveolar exposure from a trigger is known to result in release of signaling factors that also activates the endothelial barrier and increase permeability and paracellular gap formations (Kuebler et al., 2010; Bärnthaler et al., 2017). Therefore, in our studies we demonstrated disrupted endothelial barrier on NPs and PHMG exposure, whereas with FL treatment we observed an increase in CD31 (or Pecam) expression signifying an onset of the barrier recovery process. Moreover, increased vWF levels was observed on exposure to PHMG on-chip which is in line with current research findings confirming that elevated vWF amount is associated with inflammation and emphysema pathogenesis (Harrison et al., 2017; Langholm et al., 2020). It signifies that apical epithelial exposure of the NPs and toxic PHMG were able to induce a much deeper basal effect inciting endothelial activation and damage in our cellular models on-chip. FL treatment on-chip was able to reduce expression of inflammation and epithelial damage-associated genes, however it could not recover barrier damage induced by PHMG. One reason could be the small treatment window of 24 h which was not sufficient to recover major loss of barrier integrity by PHMG or booster doses of inhaled FL might be required to overcome such major damage (Kortekaas Krohn et al., 2019; Sibinovska et al., 2022).

Notably, we have found an immense effect of three-dimensional CS in our models. ALI along with CS culture conditions has a tremendous influence on the alveolar barrier and made it highly sensitive to the exposed NPs and PHMG. From our previous study (Sengupta et al., 2022) we had observed changes with the actin fibers on CS, which might hint towards a cytoskeletal rearrangement which facilitates better sensitivity to the exposed toxicants. Recent studies have shown convincing evidence that cell-ECM interactions play an important role for particle uptake kinetics and subsequent inflammation (Augustine et al., 2020; Engin et al., 2017; A; Lee et al., 2022). On the other side, abnormal ECM remodeling is a hallmark feature for COPD, asthma and fibrosis (Ito et al., 2019; Dekkers et al., 2021; Migulina et al., 2022). Therefore, it will be important to investigate the use of native lung-derived ECM hydrogels (Nizamoglu et al., 2022) along with CS in lung-on-chips to investigate inhalation toxicity. In another recent publication, authors demonstrated that under 2D linear strain, uptake of 100 nm particles was highly increased (Doryab et al., 2021) where endocytosis and cytoskeletal remodeling might play a prominent role (Freese et al., 2014; Augustine et al., 2020).

The presence of surfactant in the *in vivo* distal lung circuit for inhaled NPs is another crucial aspect to consider. Upon reaching the deep lung, NPs tend to initially interact with the lung surfactant film, which is a membrane-based lipid-protein complex secreted by alveolar cells. This film plays an important role as it insulates the respiratory ALI and can affect the toxicity of NPs (Olmeda et al., 2020). Based on the charge of NPs, they can either attach to the lipid heads or deplete the proportion of lipids. Therefore, in all cases there is an impact on the surface tension which greatly influences the interaction and translocation of NPs through the surfactant film (Radiom et al., 2020; Hidalgo et al., 2017; Bai et al., 2020). Thus, using *in vitro* ALI models with a thin layer of surfactant at the top is the recommended approach when studying toxicity of NPs (Schleh et al., 2013). Hence, it would be important for future studies to include pulmonary surfactant preparation containing saturated and unsaturated phospholipids and sufficient concentration of SP-B and SP-C proteins when performing toxicity studies using such lung-on-chip models (Pérez-Gil, 2022).

Moreover, current studies with tobacco smoke, which is known to be the major risk factor for COPD (cdc.gov) have indicated that CS plays a complex role in activating a mechano-inflammation response in cells on exposure to cigarette smoke and extract (Mondoñedo et al., 2020; Nossa et al., 2021). Hence, it will be interesting to examine the precise role of ALI and CS in toxicant uptake and reaction to cigarette smoke in future lung inhalation models.

## Conclusion

Comprehensively, the Cloud α AX12 platform recapitulates critical parameters of inhalation toxicity in the lung epithelium such as deposition kinetics, reproducible cytotoxic and barrier disruption effects in cell models of varying complexities along with deregulated gene and protein regulation representative of an inflamed barrier. NPs like TiO2 and ZnO when exposed to cells on-chip induce an inflammatory cascade resulting in barrier damage and reduced cell viability in both alveolar and bronchial cell models. Also, aerosolized PHMG induced significant cytotoxic response including reduced barrier integrity, EMT and increased expression of inflammatory cytokines in mono, co- and tri-cell culture models on-chip. Moreover, we have demonstrated that nebulized FL corticosteroid was strongly able to diminish PHMG-induced toxic effects including EMT and inflammation. Therefore, with the Cloud α AX12 we could establish three different exposure models with varying multicellular complexities, where a crucial physiological impact of ALI and CS combined culture conditions could be conceived.

The Cloud α AX12 platform thereby allows for reproducible testing conditions along with simple user-friendly handling which is crucial for development of inhaled medicines and hazard assessment studies. Overall, our results strongly advocate in favor of this inhalation lung-on-chip model, and it will prove to be of high value for pre-clinical and precision medicine studies to serve as a suitable alternative for use of animal models in inhalation research.

## Data Availability

The original contributions presented in the study are included in the article/Supplementary Material, further inquiries can be directed to the corresponding author.

## Abbreviations

COPD, Chronic obstructive pulmonary disease; ALI, air-liquid interface; CS, cyclic stretch; NPs, nanoparticles; TiO2, titanium dioxide; ZnO, zinc oxide; PHMG, polyhexamethylene guanidine; FL, fluticasone propionate; 3D, three-dimensional; ECs, endothelial cells; AMs, alveolar macrophages; AT1, alveolar type 1 cells; AT2, alveolar type 2 cells; AXhAEpC, Primary human alveolar epithelial cells; AXiAECs, primary cell-derived immortalized alveolar epithelial cell line; d-THP1, differentiated THP-1 cells; TER, transepithelial barrier; tri-cell culture, triple-cell culture; ECM, extracellular matrix; QCM, quartz crystal microbalance; OR, Output Rate; SBL, suspension base liquid; h, hours; mins, minutes; PMA, phorbol 12-myristate 13-acetate; LDH, Lactate Dehydrogenase; ROS, reactive oxygen species; fc, fold change; EMT, epithelial-mesenchymal transition.
