# Erratum: A multiplex inhalation platform to model *in situ* like aerosol delivery in a breathing lung-on-chip

**DOI:** 10.3389/fphar.2023.1229313

**Published:** 2023-06-05

**Authors:** Frontiers Production Office

**Affiliations:** Frontiers Media SA, Lausanne, Switzerland

**Keywords:** lung-on-chip, inhalation therapeutics, nanoparticles, aerosolized drug delivery, cyclic stretch, air-liquid interface, COPD, toxicity assessments

Due to a production error, the corresponding author was erroneously changed. The corresponding author is Arunima Sengupta, email: arunima.sengupta@unibe.ch.

The publisher apologizes for this mistake. The original version of this article has been updated.

