# Supplementary material for: A multiplex inhalation platform to model in situ like aerosol delivery in a breathing lung-on-chip
Source: Front Pharmacol. 2023 Mar 6;14:1114739. doi: 10.3389/fphar.2023.1114739 (PMC10029733; doi:10.3389/fphar.2023.1114739)
Supplement: Supplementary file 1 [file DataSheet1.pdf]

Supplementary Information

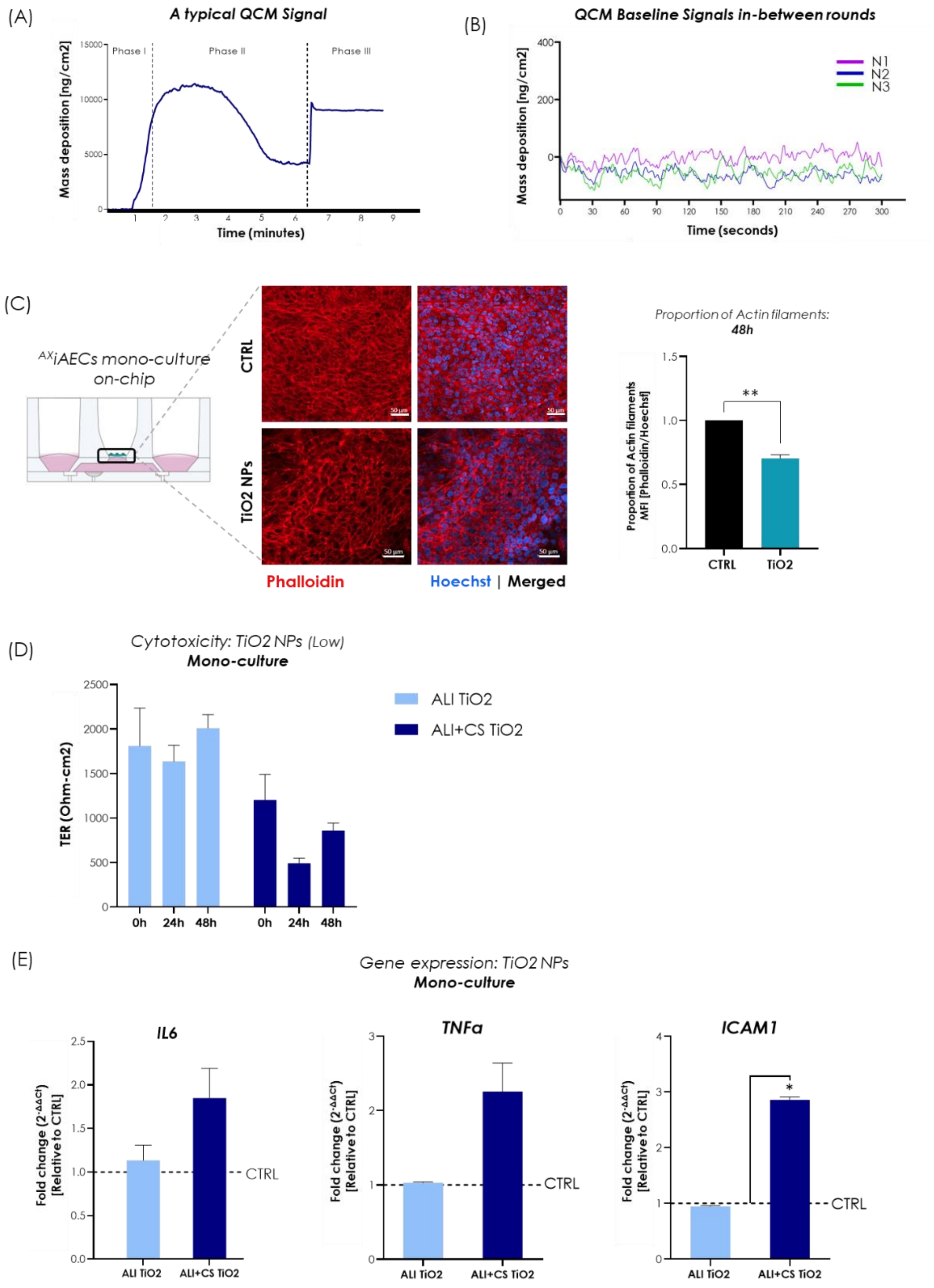

Fig. S1. TiO2 NPs exposure reduce actin filaments and increase inflammation markers in AXiAECs on-chip (A) The typical deposition profile recorded by the QCM during a nebulization experiment with the Cloud α AX12 showing the three typical phases for dose deposition. (B) Baseline QCM 6 signal measurement for three independent rounds (n=3) from the start of nebulization until the end of sedimentation (5 mins). (C) Representative immunofluorescent stainings from control (CTRL) and TiO2 NPs exposed AXiAECs on-chip. Scale bar here is 50µm. Quantitave analysis was performed by

normalizing the mean-fluorescent intensity (MFI) of Phalloidin (staining for F-actin fibers, in red) with Hoechst nuclei intensity (in blue). Data are represented as mean ± SEM (N=3; ROI = 4 per conditions per N). (D) TER was measured from untreated and low concentration TiO2 NPs (0.021µg/cm2) exposed cells under ALI and ALI+CS conditions at 0h before exposure, 24h and 48h after TiO2 NPs exposure (N=2; n=4/timepoint). (E) mRNA was harvested from control and TiO2 high concentration exposed cells (ALI and ALI+CS) at 48h exposure timepoint. qPCR studies were performed with N=2; n=2/conditions and exposure significance were measured in relation to CTRL cell values. Data are represented here as mean ± SEM.

Section 1: *in vitro* dose correlation

The translation from *in vitro* dosing of inhaled corticosteroids used in this study to the daily inhaled dosing of a patient with a respiratory condition was approached by a mathematical approximation. Thorrson et al. in 2001 demonstrated the relative deposition difference of FL in pressurized metered-dose Inhaler (pMDI) and a Diskus inhaler. A pMDI is an inhaler in which a powdered drug is pressurized together with a propellant gas inside a container. By actuation of the inhaler the drug is released aerosolized. A Diskus inhaler on the other hand is a non-pressurized inhaler which is actuated by breath. The inhalation flow aerosolizes the metered drug dose into the respiratory tract. Calculations of lung deposition were performed by considering the ratio of substance remaining in the mouth after the use of the inhaler, systemic bioavailability, plasma cortisol concentration, clearance, halftime, mean residence time, mean absorption time and volume of distribution at steady state (Thorsson et al., 2001). It was concluded that on average 20% and 12% of the nominal dose per inhalation was deposited in the lung for pMDI and Diskus drug delivery respectively. By reverse calculation of the dosage used on the lung-on-chip, assuming an average alveolar surface in the distal lung is about 70-100m^2 (Fröhlich et al., 2016) using the manufacturer (Vitrocell) provided deposition calculations per cm^2 of nebulized substance, an *in vivo* estimation of the dosage was performed. A factor of 0.85 was added to the calculation to account for the 15% deviation in mass deposition within the Cloud α AX12. The area within one cellular well of the AX12 plate was nebulized with 300µL of a Fluticasone propionate concentration, this was extrapolated to an adult healthy average lung surface area to determine the estimated mass deposited. Depending on the inhaler device the nominal dose to deposited mass ratio of the inhaled corticosteroid (Fluticasone propionate) with concentrations of 100nM and 500nM was obtained as:

$$\frac{Dose_{in-vitro} \times 0.85 \times lung\ surface\ area}{Ratio_{nominal\ dose\ (Inhaler)}} \approx Nominal\ dose[inhaler]$$

$$100nM\ Diskus \rightarrow \frac{0.0000747\ \frac{\mu g}{cm^2} \times 0.85 \times (7\ to\ 10) \times 10^5\, cm^2}{0.12} \approx 370.4\mu g - 529.1\mu g$$

$$100nM\ pMDI \rightarrow \frac{0.0000747\ \frac{\mu g}{cm^2} \times 0.85 \times (7\ to\ 10) \times 10^5\, cm^2}{0.20} \approx 222.2\mu g - 317.8\mu g$$

$$500nM\ Diskus \rightarrow \frac{0.000374\ \frac{\mu g}{cm^2} \times 0.85 \times (7\ to\ 10) \times 10^5\, cm^2}{0.12} \approx 1854.0\mu g - 2649.2\mu g$$

$$500nM\ pMDI\ \rightarrow \frac{0.000374\ \frac{\mu g}{cm^2} \times 0.85\ \times (7\ to\ 10) \times 10^5\, cm^2}{0.20} \approx 1112.6\mu g\ -\ 1589.5\mu g$$

Global initiative for asthma has recommended the use of 100-250nM as total daily low dose, >250-500 as medium and above 500nM as high total daily dose (Global Initiative for Asthma 2022). Therefore, our 100nM FL dose correlates to medium recommended daily dosing in adults, whereas 500nM corresponds to high daily dose for an adult.

Next, to approximate the deposition of NPs *in vivo* from *in vitro* gathered data, a similar approach was taken. Paur et al. and colleagues estimated a normal and a worst-case concentration level delivered to the lungs in 24h and over the entire lifetime (Paur et al., 2011). Their Approximation was based on a total lifetime dose under ambient conditions of 6.6 mg/cm2 (in 80 years). Under highly polluted conditions, or for factory workers a lifetime dose of 450 mg/cm2 was expected. This is a deposition of 0.005ug/cm2 per hour deposited in a healthy human lung. As these calculations are based on an average deposition efficiency of 30%, this value must be adapted to the NPs deposition efficiency tested. Deposition efficiency was estimated from the NPs airborne diameter size (Geiser & Kreyling, 2010). Hence, for ZnO NPs, deposition efficiency was around 15-50% and for TiO2 NPs was 10-20%. The dose correlation was then performed using the following equation:

Diameter of ZnO: 30–150 nm (European commission, 2015)

Diameter of TiO2: 100–250nm (TDMA, 2021)

$$dose_{in\ vitro} / \frac{dose_{worst-case\ lifetime}}{hour} \times \frac{0.30}{deposition\ efficiency_{NP}}$$

$$\approx nr\ of\ hours\ spent\ in\ highly\ poluted\ area$$

$$Deposition\ efficiency\ of\ ZnO: 15 - 50\%$$

$$\frac{100ug}{ml} ZnO \rightarrow \frac{0.1494\ \mu g/cm^2}{0.0050\ \frac{\mu g/cm^2}{h}} \times \frac{0.30}{0.15\ to\ 0.50} \approx \underline{\underline{18h - 60h}}$$

$$Deposition\ efficiency\ of\ TiO_2: 10 - 20\%$$

$$\frac{100ug}{ml} \rightarrow \frac{0.1494\ \mu g/cm^2}{0.0050\ \frac{\mu g/cm^2}{h}} \times \frac{0.30}{0.10\ to\ 0.20} \approx \underline{\underline{45h - 90h}}$$

It shows that different NPs have a diameter size dependent range of h spent in a highly polluted area. As the dispersion of ZnO is very broad the deposition ratio is likewise and therefore the time calculated for the exposure to ZnO. TiO2 has a much narrower deposition efficiency (Pedrini et al., 2021) which leads to an increased average time spent in a highly polluted area to get the same deposition concentration as ZnO in the lung.

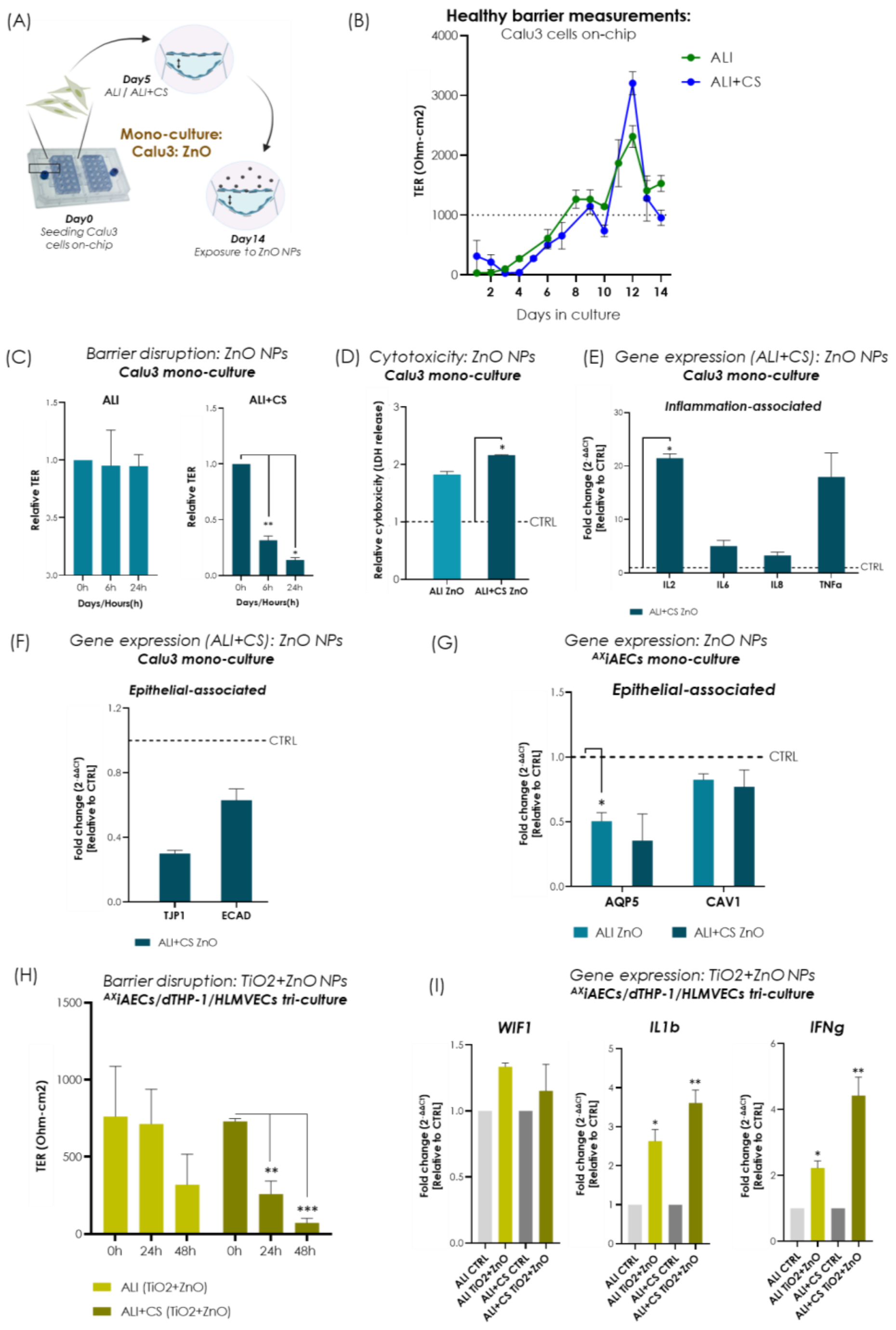

Fig. S2. ZnO NPs alone or in combination with TiO2 NPs cause barrier damage and epithelial and inflammation gene deregulation (A) timeline for Calu3 cell seeding on-chip and exposure to ZnO NPs. (B) TER (Ohm-cm2) was measured

from healthy Calu3 cells on-chip from day 2 until day 14 (N=3; n=6/timepoint). (C) TER (Ohm-cm2) was measured from ZnO NPs nebulized Calu3 cells at 0h (before exposure) and 6h and 24h after exposure under ALI and ALI+CS culture conditions (N=2; n=3-5/timepoint/conditions). (D) LDH measured under ALI and ALI+CS Calu3 cells exposed to ZnO NPs in relative to CTRL cells (N=2; n=4). (E) mRNA was harvested from ALI+CS conditions at 24h timepoint and measured for inflammatory markers like IL2, IL6, IL8 and TNFα. Significance was calculated in relation to CTRL cell expression values (N=2; n=4) and (F) mRNA was harvested from ALI+CS Calu3 cells on-chip at 24h timepoint and measured for epithelial related genes like TJP1 and ECAD (N=2; n=4). Significance was calculated in relation to CTRL healthy cell expression levels. (G) mRNA isolated from CTRL and ZnO exposed ^{AX}iAECs mono-cell culture on-chip (in ALI and ALI+CS) at 48h exposure timepoint. qPCR studies were performed with N=2; n=2/conditions and exposure significance were measured in relation to CTRL samples. (H) TER readings were recorded from ^{AX}iAECs/d-THP1s/HLMVECs tri-cell culture on-chip before (0h) and 24h and 48h after exposure with TiO2+ZnO NPs mixture (n=4-6/conditions/time-point). (I) Gene expression was analyzed for WIF1, IL1b and IFNg from both ALI and ALI+CS samples at 48h (n=4/conditions). Data are shown as mean ± SEM.

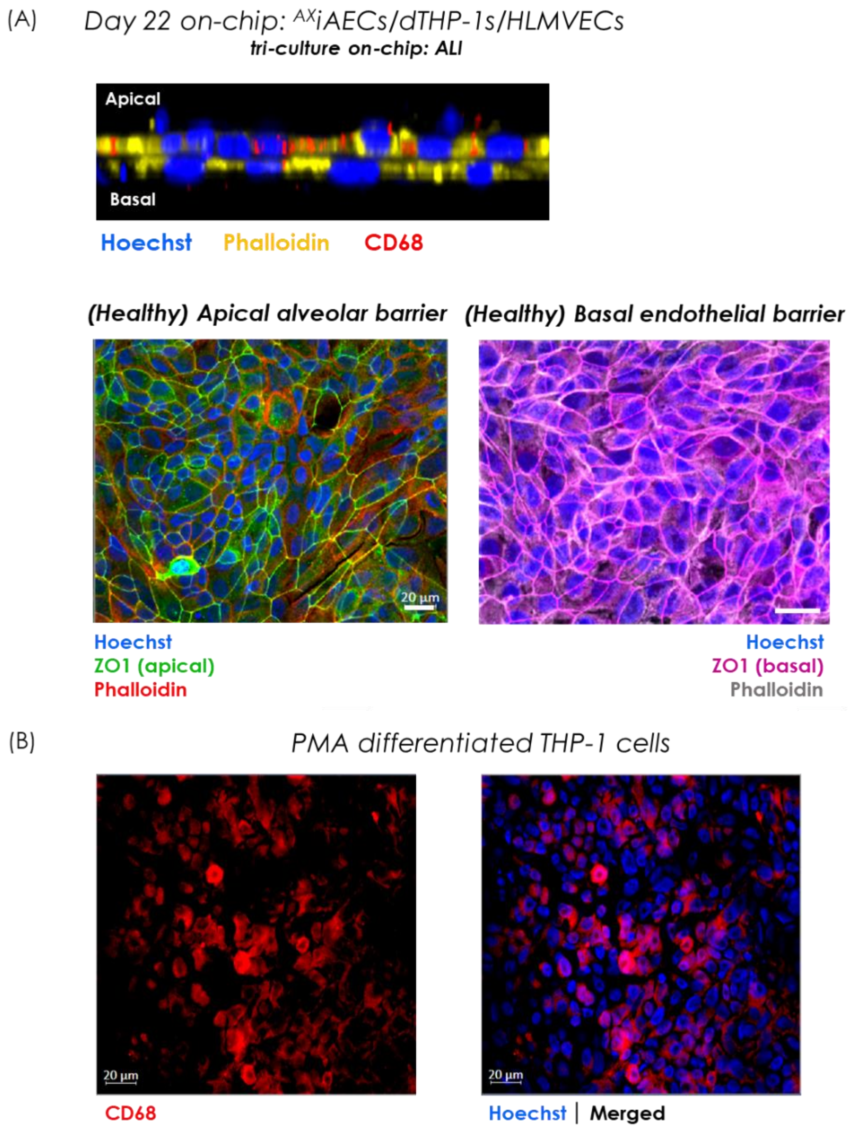

Fig. S3. Characterization of triple-cell culture model on-chip (A) In top, XZ axis view of the tri-cell culture model on-chip under ALI condition. Nuclei is stained with Hoechst on apical and basal side, Phalloidin is stained in yellow and CD68+ differentiated THP-1 macrophages are stained in red on the apical side. In bottom, apical alveolar and basal endothelial barrier is shown. The tight junction, ZO1 is stained in green for apical and magenta for basal cells and the nuclei with

Hoechst in blue. Phalloidin is stained in red for apical and light grey for basal cells. Scale bar is 20µm. (B) Representative staining confirms differentiation of THP-1 monocytes to d-THP1 macrophages with positive expression of CD68 marker (in red). Scale bar here is 20µm.

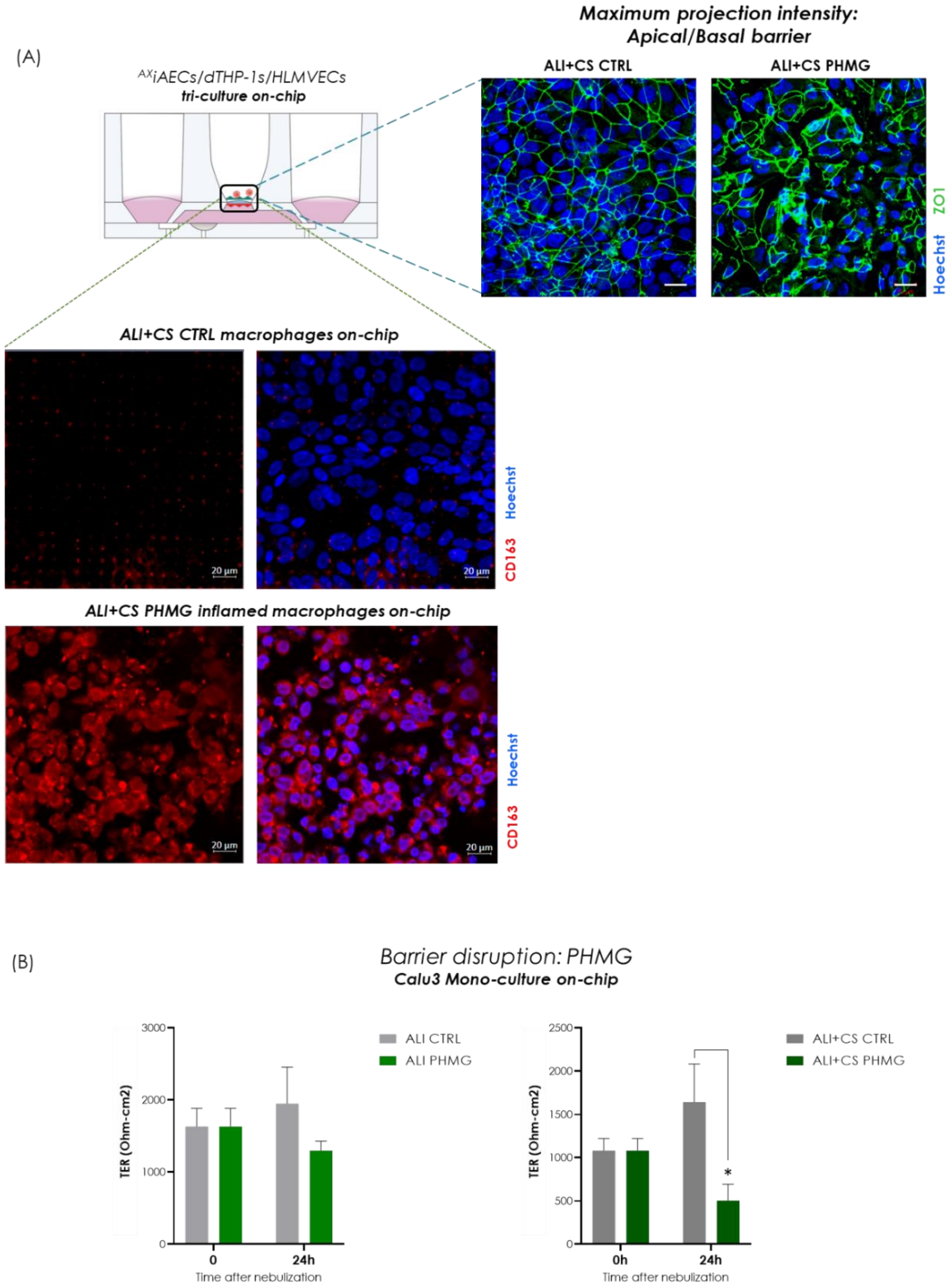

Fig. S4. Characterization of PHMG-induced cell barrier damage (A) Maximum projection intensity images were obtained from confocal z-stacks of alveolar and basal cells in the tri-cell culture setup. Control and PHMG exposed cells on-chip were stained with ZO1 (in green), CD163 (in red) and nuclei with Hoechst (in blue). (B) TER (Ohm-cm2) was measured before (0h) and 24h after PHMG exposure to Calu3 cells on-chip in both ALI and ALI+CS conditions (N=1; n=5-6/conditions/time-point). Data are represented as mean ± SEM.

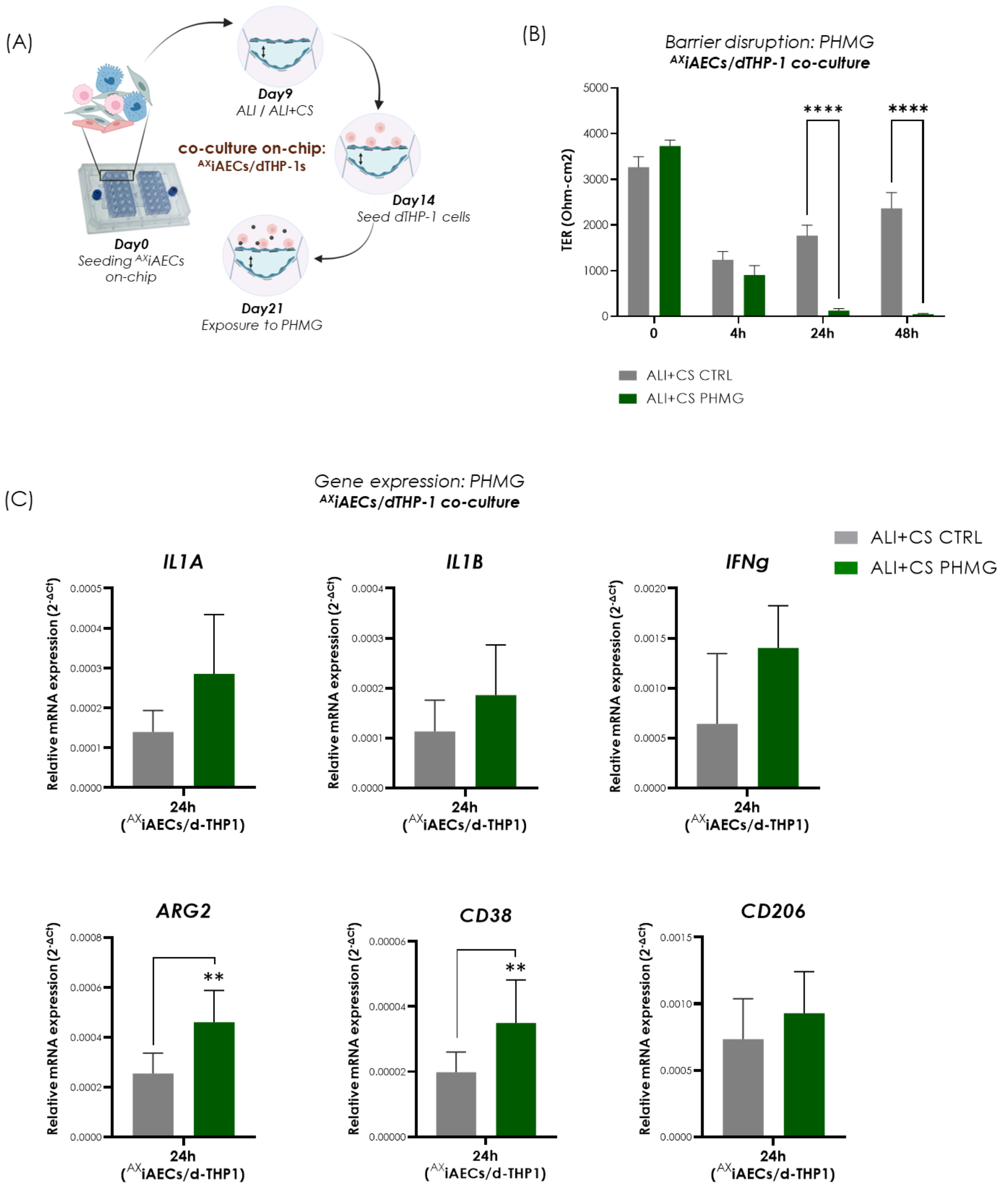

Fig. S5. Nebulized PHMG exposure in (^{AX}IAECs/d-THP1) co-culture on-chip incite barrier gaps and inflammation (A) Overall schematic and timeline for ^{AX}IAECs/d-THP1 co-culture seeding and PHMG exposure in the AX12 plate. (B) TER (Ohm-cm2) was measured for cells in ALI+CS culture conditions before (0h) and 4h, 24h and 48h after exposure to PHMG (N=3; n=12). (C) qPCR studies were performed with mRNA extracted from ALI+CS cells (from 24h time-point) ± PHMG exposure (N=3;n=8). Data are represented as mean ± SEM.

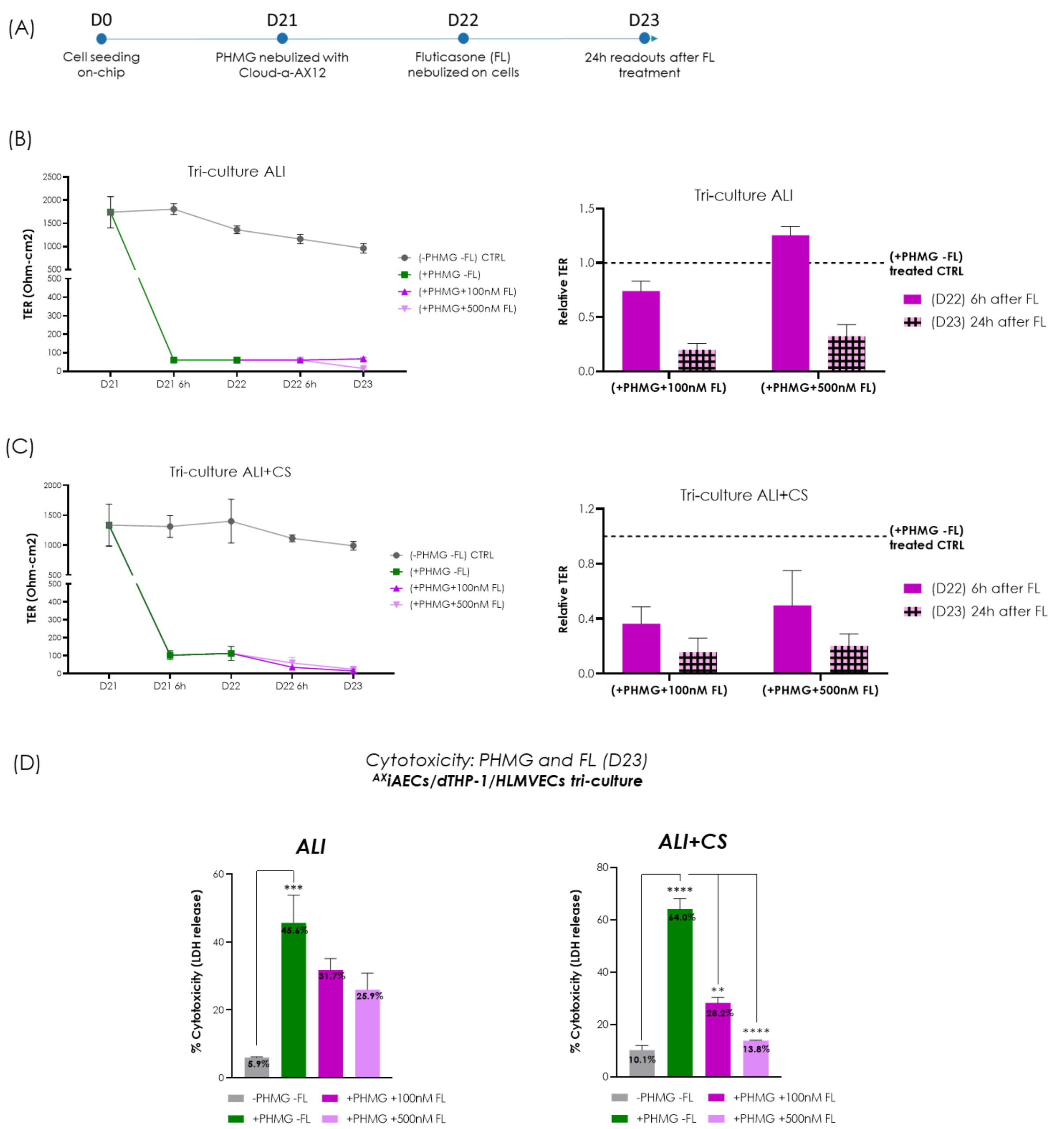

Fig. S6. Nebulized Fluticasone (FL) treatment effect on barrier recovery and cytotoxicity triggered by PHMG induction on-chip (A) Overall timeline for cell seeding starting at Day 0 (D0) to PHMG exposure on D21 and FL treatment on D22 in the AX12 plate. (B) Time-series TER profile (Ohm-cm2) was measured for cells in ALI and (C) ALI+CS culture conditions, including before induction (0h) to 6h and 24h after PHMG exposure and until 24 h after FL treatment (N=2; n=6). (D) Cytotoxicity was calculated from LDH levels for ALI and ALI+CS samples (N=1; n=3/condition). Data are represented as mean ± SEM.

Gene name	Forward primer (5’-3')	Reverse primer (5’-3')
IL2	AC AAG AAC CCG AAA CTG ACT CG	CA TGA AGG TAG TCT CAC TGC C
IL1a	GA TGC CTG AGA TAC CCA AAA CC	CA AAG CAC ACC CAG TAG TCT

IL1b	CA CGA TGC ACC TGT ACG AT	AC CAA GCT TTT TTG CTG TGA GT
IL6	GG AGA CTT GCC TGG TGA AA	TC AGG GGT GGT TAT TGC AT
IL8	GC ACT CCT TGG CAA AAC TG	GG AAG GAA CCA TCT CAC TG
TNFa	TG AGC ACT GAA AGC ATG ATC	AG GGC TGA TTA GAG AGA GGT
ICAM1	GC CGG CCA GCT TAT ACA C	AG ACA CTT CAG CTC GGG CA
AQP5	CC ACC TTG TCG GAA TCT ACT	GC TCA TAC GTG CCT TTG ATG
CDH1	GA GAG CTA CAC GTT CAC GG	GA GAG CTA CAC GTT CAC GG
CAV1	AC GAT GAC GTG GTC AAG ATT G	CC AAA TGC CGT CAA AAC TGT
TJP1	GA AAC CCG AAA CTG ATG CTA TGG	AC TGG CTG GCT GTA CTG TGA G
WIF1	AG GTT GGC ATG GAA GAC AC	TA AGT GAA GGC GTG TGC TG
MUC1	CT GCT TCT ACT CTG GTG CAC AA	TT GAG AAT GGA GTG CTC TTG CT
IFNg	GC TCT GCA TCG TTT TGG GTT	TT CCA TTA TCC GCT ACA TCT GAA
ARG2	AG CTG GCT TGA TGA AAA GGC	CG TGG ATT CAC TAT CAG GTT GT
CD38	AA CTC TGT CTT GGC GTC AGT	CC ATA CAC TTT GGC AGT CTA CA
CD206	CC GGG TGC TGT TCT CCT A	CA GTC TGT TTT TGA TGG CAC
VE-cad	TC CAC AAA GCT CGG CCC TGG	GG CCC AGG AAG GCT CCC AA
PECAM	TG CCA GTC CGA AAA TGG AAC	TT CAT CCA CCG GGG CTA TC
vWF	CT TGA ATC CCA GTG ACC CTG A	GT TCC GAG ATG TCC TCC ACA T
HPRT	GA CTT TGC TTT CCT TGG TCA GG	TC TGG CTT ATA TCC AAC ACT TCG

Table. S1. Primer sequences
